# Predictive and prognostic potential of pretreatment ^68^Ga-PSMA PET tumor heterogeneity index in patients with metastatic castration-resistant prostate cancer treated with 177Lu-PSMA

**DOI:** 10.3389/fonc.2022.1066926

**Published:** 2022-12-08

**Authors:** Majid Assadi, Reyhaneh Manafi-Farid, Esmail Jafari, Ahmad Keshavarz, GhasemAli Divband, Mohammad Mobin Moradi, Zohreh Adinehpour, Rezvan Samimi, Habibollah Dadgar, Narges Jokar, Benjamin Mayer, Vikas Prasad

**Affiliations:** ^1^ The Persian Gulf Nuclear Medicine Research Center, Department of Nuclear Medicine, Molecular Imaging, and Theranostics, Bushehr Medical University Hospital, School of Medicine, Bushehr University of Medical Sciences, Bushehr, Iran; ^2^ Research Center for Nuclear Medicine, Shariati Hospital, Tehran University of Medical Sciences, Tehran, Iran; ^3^ IoT and Signal Processing Research Group, ICT Research Institute, Faculty of Intelligent Systems Engineering and Data Science, Persian Gulf University, Bushehr, Iran; ^4^ Khatam PET-CT center, Khatam Hospital, Tehran, Iran; ^5^ Department of Medical Radiation Engineering, Shahid Beheshti University, Tehran, Iran; ^6^ Cancer Research Center, RAZAVI Hospital, Imam Reza International University, Mashhad, Iran; ^7^ Institute of Epidemiology and Medical Biometry, Ulm University, Ulm, Germany; ^8^ Department of Nuclear Medicine, University Hospital Ulm, Ulm, Germany

**Keywords:** radiomics, 177Lu-PSMA, 68Ga-PSMA, PET-CT, metastatic castration-resistant prostate cancer (mCRPC)

## Abstract

**Introduction:**

This study was conducted to evaluate the predictive values of volumetric parameters and radiomic features (RFs) extracted from pretreatment 68Ga-PSMA PET and baseline clinical parameters in response to 177Lu-PSMA therapy.

**Materials and methods:**

In this retrospective multicenter study, mCRPC patients undergoing 177Lu-PSMA therapy were enrolled. According to the outcome of therapy, the patients were classified into two groups including positive biochemical response (BCR) (≥ 50% reduction in the serum PSA value) and negative BCR (< 50%). Sixty-five RFs, eight volumetric parameters, and also seventeen clinical parameters were evaluated for the prediction of BCR. In addition, the impact of such parameters on overall survival (OS) was evaluated.

**Results:**

33 prostate cancer patients with a median age of 69 years (range: 49-89) were enrolled. BCR was observed in 22 cases (66%), and 16 cases (48.5%) died during the follow-up time. The results of Spearman correlation test indicated a significant relationship between BCR and treatment cycle, administered dose, HISTO energy, GLCM entropy, and GLZLM LZLGE (p<0.05). In addition, according to the Mann-Whitney U test, age, cycle, dose, GLCM entropy, and GLZLM LZLGE were significantly different between BCR and non BCR patients (p<0.05). According to the ROC curve analysis for feature selection for prediction of BCR, GLCM entropy, age, treatment cycle, and administered dose showed acceptable results (p<0.05). According to SVM for assessing the best model for prediction of response to therapy, GLCM entropy alone showed the highest predictive performance in treatment planning. For the entire cohort, the Kaplan-Meier test revealed a median OS of 21 months (95% CI: 12.12-29.88). The median OS was estimated at 26 months (95% CI: 17.43-34.56) for BCR patients and 13 months (95% CI: 9.18-16.81) for non BCR patients. Among all variables included in the Kaplan Meier, the only response to therapy was statistically significant (p=0.01).

**Conclusion:**

This exploratory study showed that the heterogeneity parameter of pretreatment 68Ga-PSMA PET images might be a potential predictive value for response to 177Lu-PSMA therapy in mCRPC; however, further prospective studies need to be carried out to verify these findings.

## Introduction

Metastatic castration-resistant prostate cancer (mCRPC) is associated with a poor patient prognosis (1). Cancer spreads throughout the body, often to bones, with spatial and temporal variations that make treatment challenging (2). Due to the highly heterogeneous nature of prostate cancer, a challenge for clinical disease management is that patients from different ethnic and geographical backgrounds have different genomic alterations, suggesting that prostate carcinogenesis proceeds along distinct pathways (3). Additionally, solid biopsy cannot characterize the spatial heterogeneity caused by the evolution of the disease.

Positron emission tomography (PET) scans are becoming increasingly important for the evaluation of response prediction using tumor textural analysis (a measurement of spatial heterogeneity) (4, 5). Considering that prostate-specific membrane antigen (PSMA)-PET/computed tomography (PET/CT) is a vital method for diagnosing and treatment planning in advanced prostate cancer (6), obtaining additional information using radiomic features (RFs) is a highly desirable approach, especially when it comes to treatment planning (7). Radiomics involves the transformation of digitally encrypted medical images containing information related to tumor pathophysiology into mineable high-dimensional data. Clinical-decision support systems can use this information *via* quantitative image analyses to improve medical decisions (8).

Radioligand therapy involves binding therapeutic radiopharmaceuticals to specific receptors or antigens on tumor cell surfaces in order to produce direct tumoricidal effects with minimal/manageable side effects (9). According to the latest studies, treatment with [177Lu] Lu-PSMA-617 is effective and well tolerated and about 70% of the patients experience positive outcomes (10–13). It was recently found that radiopeptide therapy with 177Lu-PSMA has a non-responder rate of about 30% (no PSA decline) (12, 14, 15). And a big challenge is why all the patients with high uptake on PSMA PET fail on treatment using 177Lu-PSMA.

Therefore, this study was conducted to investigate the role of RFs and volumetric parameters extracted from pre-treatment 68Ga-PSMA PET-CT images as well as baseline clinical factors in predicting response to 177Lu-PSMA therapy and estimating the overall survival (OS) of mCRPC patients.

## Materials and methods

### Patients

In this multicenter retrospective study, the mCRPC patients undergoing 177Lu-PSMA therapy and performed 68Ga-PSMA PET-CT within one month before Lu-PSMA therapy were enrolled from September 2017 to January 2022. Inclusion criteria included confirmed mCRPC with positive PSMA expression according to the 68ga-PSMA PET-CT. The exclusion criteria included white blood cell (WBC) count less than 2000/µl, platelet count less than 60000/µl, creatinine more than 2 mg/dl, and significant impairment of bone marrow, liver, and kidneys. The available clinical data of all mCRPC patients were collected and documented.

### Ga-PSMA PET-CT

In this study, data were collected from three different centers with three PET-CT systems (center 1: Siemens Biograph 6 Truepoint; center 2: Siemens Biograph 6 Truepoint, center 3: Siemens Biograph mCT). Image acquisition was performed about 60 minutes after intravenous injection of 100-150 MBq 68Ga-PSMA. A low-dose CT (50 mAs, 130 kVp) was done followed by a PET scan with 3-4 minutes per bed. The CT data were reconstructed with 5 mm slices and 512 to 512 matrices. The PET data were reconstructed with 128 to 128 matrices using the iterative attenuation-weighted ordered subset algorithm (2 iterations; 16 subsets). CT data were used for scatter and attenuation correction.

### 177Lu-PSMA-617 therapy

Commercially available radiolabeled 177Lu-PSMA-617 was obtained from Pars Isotope Co., Iran. Following intravenous injection of 1000 ml normal saline, 3.7-7.4 GBq 177Lu-PSMA-617 was injected intravenously. The time interval between each cycle was 6-8 weeks. Hematological indexes, liver function, renal function, alkaline phosphatase (ALP), and PSA were checked at baseline within a few days before therapy and repeated monthly until 2 months after the last cycle.

Whole body scan (and SPECT if needed) was performed at 24 and 48 hours after radiotracer injection to evaluate radiotracer distribution. The treatment was stopped if no or minimal uptake of PSMA in the lesions was observed. Furthermore, if inadequate organ function was observed (platelets >75,000 × 109/L, neutrophil >1.5 × 109/L), the treatment was discontinued until the function returned to the desired level.

### Treatment response

The biochemical response (BCR) was defined as a 50% or more reduction in the PSA level compared to the baseline. Otherwise, the patient was considered as no response.

Overall survival (OS) was measured as the time interval between the beginning of the therapy and death.

### Image analysis

For each scan, an experienced nuclear medicine physician identified and segmented all the pathological hotspots semi-automatically using 3D Slicer version 4.11 (http://www.slicer.org) (16). All radiomic features (RF) were extracted using the LIFEx package (http://www.lifexsoft.org) (17). Resampling was done for RF extraction, (voxel size: 2*2*2 mm^3^). The number of grey levels for intensity discretization was 64, and normalization was also performed. RF extraction was performed for lesions with volumes more than 3 cm^3^. Totally, 65 RFs were extracted from PET images including 28 first-order, 5 shapes, 7 gray-level co-occurrence matrices (GLCM), 11 grey-level run-length matrices (GLRLM), 3 neighboring gray-level dependence matrix (NGLDM), and 11 grey-level zone length matrix (GLZLM) features (**Table 1**). In addition, eight quantitative parameters were calculated including D_max_ as the distance between two lesions furthest apart, total PSMA tumor volume (PSMA-TV), PSMA-TV_max_, PSMA-TV_mean_, maximum total lesion PSMA (TL-PSMA_max_), TL-PSMA_mean_, total TL-PSMA, and tumor-to-liver ratio (TLR) as SUV_max_ of tumor over SUV_max_ of the healthy liver tissue.

**Table 1:** Extracted radiomics features.

Conventional (n=11)	SUVbwmin SUVbwmean SUVbwstd SUVbwmax SUVbwQ1 SUVbwQ2 SUVbwQ3 SUVbwSkewness SUVbwKurtosis SUVbwExcessKurtosis TLG
Discretized (n=11)	SUVbwmin SUVbwmean SUVbwstd SUVbwmax SUVbwQ1 SUVbwQ2 SUVbwQ3 SUVbwSkewness SUVbwKurtosis SUVbwExcessKurtosis TLG
Histogram (n=6)	Entropy_log10 Entropy_log2 Energy AUC-CSH
Shape	Volume (mL) Volume (voxel) Sphericity Surface Compacity
GLCM (n=7)	Homogeneity Energy Contrast Correlation Entropy_log10 Entropy_log2 Dissimilarity
GLRLM (n=11)	SRE LRE LGRE HGRE SRLGE SRHGE LRLGE LRHGE GLNU RLNU RP
NGLDM (n=3)	Coarseness Contrast Busyness
GLZLM (n=11)	SZE LZE LGZE HGZE SZLGE SZHGE LZLGE LZHGE GLNU ZLNU ZP

SUV, standardized uptake value; TLG, total lesion glycolysis; GLCM, grey-level co-occurrence matrix; GLRLM, grey-level run length matrix; NGLDM, neighborhood grey-level difference matrix; GLZLM, grey-level zone length matrix; AUC-CSH, area under the curve of the cumulative SUV-volume histograms SRE, short-run emphasis; LRE, long-run emphasis; LGRE, low gray-level run emphasis; HGRE, high gray-level run emphasis; SRLGE, short-run low gray-level emphasis; SRHGE, short-run high gray-level emphasis; LRLGE, long-run low gray-level emphasis; LRHGE, long-run high gray-level emphasis; GLNU, gray-level non-uniformity; RLNU, run length non-uniformity; RP, run percentage; SZE, short-zone emphasis; LZE, Long-Zone Emphasis; LGZE, low gray-level zone emphasis; HGZE, high gray-level zone emphasis; SZLGE, short-zone low gray-level emphasis; SZHGE, short-zone high gray-level emphasis; LZLGE, long-zone low gray-level emphasis; LZHGE, long-zone high gray-level emphasis; GLNU, gray-level non-uniformity; ZLNU, zone length non-uniformity; ZP, zone percentage.

In each class of features, the features with correlation coefficients more than 0.8 were eliminated for further analysis. After removing correlated features, 34 features remained and feature harmonization was performed using ComBat harmonization to remove the center-dependent effects on RF due to different acquisition and reconstruction parameters (18, 19).

In addition to RF, seventeen clinical factors were collected for each patient (**Table 2**).

**Table 2:** Description of the clinical parameters.

Parameters	Description
Age	–
Gleason score	Stage of cancer cells in prostate
ALP	Serum alkaline phosphatase at PSMA PET
Time from initial diagnosis	Time between the first diagnosis and PSMA PET
Creatinine	Serum creatinine at PSMA PET
Hemoglobin	Hemoglobin count at PSMA PET
Platelet	Platelets count at PSMA PET
RBC	Red blood cell count at PSMA PET
WBC	White blood cell count at PSMA PET
Radiotherapy	Prior history of radiotherapy
Chemotherapy	Prior history of chemotherapy
Prostatectomy	Prior history of prostatectomy
Initial PSA	Serum PSA at PSMA PET
Treatment cycle	The number of 177Lu-PSMA cycles
Dose	The total administered dose of 177Lu-PSMA
SGPT	Serum Glutamic Pyruvic Transaminase at PSMA PET
SGOT	Serum glutamic-oxaloacetic transaminase at PSMA PET

### Statistical analysis

MATLAB 2014a (MathWorks, Natick, MA) and IBM SPSS Statistics for Windows, version 21 (IBM Corp., Armonk, N.Y., USA) were used for data analysis. After calculating RFs, they were combined to create feature vectors by averaging the amounts of the RFs of all lesions of each patient. To evaluate RF repeatability, the intraclass correlation coefficient (ICC) was calculated for each feature, and features with coefficients more than 0.8 were considered for further analysis (20). The Spearman correlation test was used to evaluate the correlation of response to treatment with RFs and clinical parameters. Furthermore, the Mann-Whitney U test was used to assess the difference in RFs and clinical parameters between BCR and non BCR. Finally, receiver operating characteristic (ROC) curve analysis with the area-under-the-curve (AUC) and cut-off value was used to choose features and clinical parameters for the prediction of BCR. AUCs more than 0.65 and p-values less than 0.05 were considered for further analysis.

Mann–Whitney U test and Spearman test were administered to evaluate the difference and correlation between two independent groups. Support vector machine (SVM) classification with AUC was used to predict response to treatment for RF and clinical factors. Kaplan–Meier analysis and log-rank test were applied to evaluate the effect of selected features and clinical factors on OS. P-values less than 0.05 were considered as statistically significant.

## Results

Thirty-three prostate cancer patients with a median age of 69 years (range: 49-89 years) were enrolled in this retrospective study. The median serum PSA value was 40 ng/ml before therapy (range 0.29-624). The median GS was 8 ranging between 5 and 10. BCR was observed in 22 cases (66%) and 16 cases (48.5) died during the follow-up time. **Table 3** shows the baseline characteristics of the patients.

**Table 3:** Patients’ characteristics.

Characteristics	
**Age (years)** **Median (range)**	69 (49-89)
**Time from initial diagnosis (years (range))**	4 (1-20)
**PSA (ng/ml)** **Median** **Range**	40 0.29-624
**Alkaline phosphatase (U/L)** **Median** **Range**	239 101-1900
**Radical prostatectomy, n (%)** **Yes** **No**	10 (30.3%) 23 (69.7%)
**Chemotherapy/Radiotherapy** **Radiotherapy** **Chemotherapy** **Both**	2 (9.5%) 7 (33.3%) 9 (42.9%)
**Gleason Score** **Median (range)** **<7** **7** **8** **≥9**	8 (5-10) 1 (3%) 9 (27.3%) 8 (24.2%) 4 (12.2%)
**Cycle** **Median (range)**	3 (1-7)
**Dose (GBq)** **Median** **Range**	22.2 7.4-50.70

One numerical (age) and 16 categorical therapeutic clinical parameters (such as Gleason score, ECOG1, and ALP1) were used for each patient. Totally, 2517 hotspots were segmented in 33 patients (median: 43, range: 1-311).

**Figure 1:**
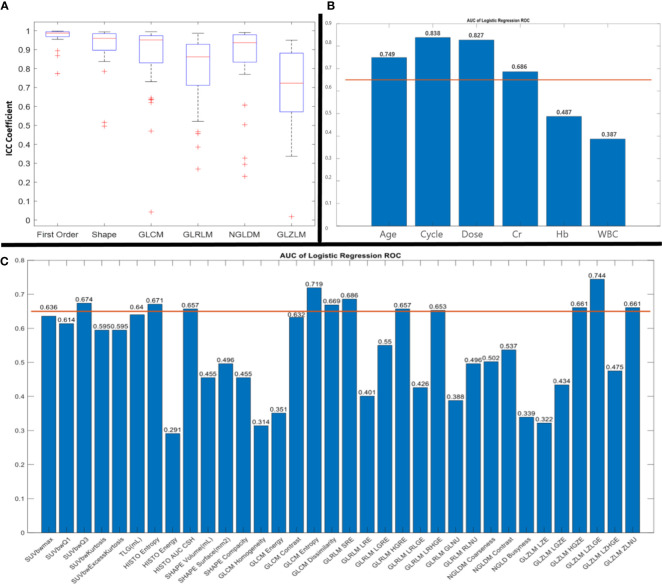
Results of intraclass correlation coefficient (ICC) **(A)** and receiver operating characteristic (ROC) curve analysis with area under the curve (AUC) of clinical parameters **(B)** and radiomic features **(C)**. AUC more than 0.65 was considered for further analysis.

According to the ICC results, of six types of radiomic features, all but GLZLM showed acceptable ICC coefficients (**Figure 1**).

The results of the Spearman correlation test indicated a significant relationship between BCR and age (r_s_: 0.416; p: 0.016), treatment cycle (r_s_: 0.56; p: 0.001), administered dose (r_s_: 0.53; p: 0.001), HISTO energy (r_s_: -0344; p: 0.05), GLCM entropy (r_s_: 0.36; p: 0.04) and GLZLM LZLGE (r_s_: -0.4; p: 0.02). In addition, according to the Mann-Whitney U test, age, cycle, dose, GLCM entropy, and GLZLM LZLGE resulted in a significant difference between BCR and non BCR patients (p<0.05).

According to the ROC curve analysis for feature selection for the prediction of BCR, GLCM entropy (AUC, 0.719; p-value, 0.043) showed acceptable results. In addition, three clinical parameters had AUC ROC more than 0.65 and p-value< 0.05 for BCR prediction including age (AUC, 0.749; p-value, 0.023), treatment cycle (AUC, 0.838; p-value, 0.002), administered dose (AUC, 0.827; p-value, 0.003) (**Figure 1**).

**Figure 2:**
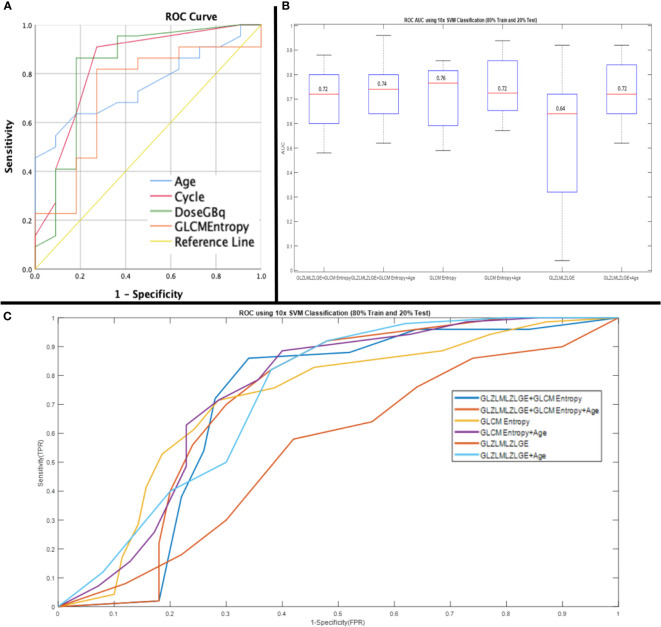
Receiver operating characteristic (ROC) curve of parameters showed significant impact on PSA response (age: AUC, 0.75; 95%CI, 0.59-0.92), (cycle: AUC,0.83; 95%CI, 0.67-0.99), (Dose: AUC, 0.82; 95%CI, 0.65-1), (GLCM entropy AUC, 0.72; 95%CI, 0.52-0.91) **(A)** and, ROC curve analysis using 10* support vector machine (SVM) classification (80% train and 20% test) for prediction of response to 177Lu-PSMA therapy (GLZLMLZLGE+GLCM Entropy: AUC, 0.72; 95%CI, 0.63-0.83), (GLZLMLZLGE+GLCM Entropy+Age: AUC, 0.74; 95%CI, 0.67-0.80), (GLCM Entropy: AUC, 0.76; 95%CI, 0.71-0.87), (GLCM Entropy+Age: AUC, 0.72; 95%CI, 0.70-0.86), (GLZLMLZLGE: AUC, 0.64; 95%CI, 0.20-0.62), (GLZLMLZLGE+Age: AUC, 0.72; 95%CI, 065-0.82) **(B, C)**.

The ideal cut-off value for GLCM entropy was 7.405 (sensitivity: 82%, specificity: 73%). The ideal cut-off values for age, dose, and cycle were 69.5 years (sensitivity: 64%, specificity: 82%), 17.95 GBq (sensitivity: 86%, specificity: 82%), and 2.5 (sensitivity: 91%, specificity: 73%), respectively. Furthermore, the ROC curve analysis for prediction of no response to therapy showed the acceptable results of GLZLM LZLGE with an ideal cut-off value of 4.375 (AUC: 0.744; p-value: 0.024, sensitivity: 100%, specificity: 50%). According to the SVM classifier, selected parameters showed acceptable AUC ROC for the prediction of response to therapy (**Figure 2**).

**Figure 3:**
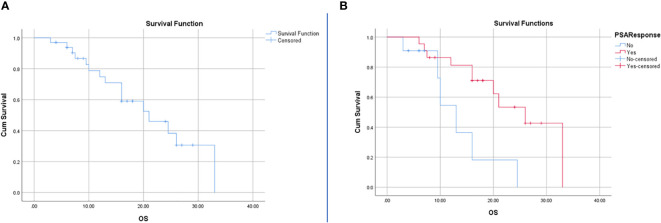
Survival analysis using Kaplan-Meier for all patients **(A)** and patients with BCR and no BCR **(B)**. The resulting P-value for the log rank test was 0.01 and hazard ratio of 0.27 with 95%CI of 0.09-0.81.

For the entire cohort, the Kaplan-Meier test revealed a median OS of 21 months (95% CI: 12.12-29.88). The median OS was 26 months (95%CI: 17.43-34.56) for BCR patients and 13 months (95%CI: 9.18-16.81) for no BCR patients. Among all variables included in the Kaplan Meier, the only response to therapy was statistically significant (p=0.01) and none of the RFs and other clinical factors achieved a p-value of less than 0.05 (**Figure 3**).

Finally, there was no significant relationship between BCR and D_max_, total PSMA-TV, PSMA-TV_max_, PSMA-TV_mean_, TL-PSMA_max_, TL-PSMA_mean_, total TL-PSMA, and TLR (p>0.05) (**Table 4**).

**Table 4:** The results of pre-treatment 68Ga-PSMA PET/CT derived quantitative parameters.

		*Dmax*	*TLR*	*TL-PSMA*	*PSMA-TV*	*PSMA-TVmax*	*PSMA-TVmean*	*TL-PSMAmax*	*TL-PSMAmean*
***No BCR***	Median	100.99	9.99	2593	483	116	19.13	452.7	69.6
	Range	63.07-176.41	1.49-11.64	69-13466	37-2942	12.93-2789	6.77-163	69-12738	28.35-747
***BCR***	Median	83.07	6.24	4909	870.5	79.61	12.42	584	81.98
	Range	47.86-149.92	0.83-42.86	86-34534	27-5522	9.8-3397	3.26-58	45.33-17513	12.9-591
***Total***	Median	90.97	6.79	4436	707	87.33	13.9	533	74.1
	Range	47.86-176.41	0.83-42.86	69-34534	27-5522	9.8-3397	3.26-163	45.33-17513	12.9-747

## Discussion

Despite the increasing number of new therapeutic agents, mCRPC remains a major challenge for clinical oncologists. Among new introduced agents, according to previous studies, 177Lu-PSMA therapy has acceptable outcomes with low toxicity in clinical practice (21, 22). However, about two-thirds of the patients treated with 177Lu-PSMA show BCR and about one-third show no BCR (23, 24). Therefore, the identification of factors associated with response to 177Lu-PSMA is important to achieve better treatment outcomes through optimizing patient selection.

Pre-treatment diagnostic 68Ga-PSMA PET-CT is traditionally used for patient selection for 177Lu-PSMA therapy; however, in most studies, response to therapy cannot be predicted with quantitative PET factors such as SUV_max_ (25–27). Nowadays, computerized diagnostics of RFs are used for the prediction of different treatment agents in several cancers (28, 29). The present study was conducted to predict response to 177Lu-PSMA therapy and also survival in mCRPC patients using clinical parameters and RFs extracted from pre-treatment 68Ga-PSMA PET-CT images. To the best of our knowledge, this study might be the first evidence assessing potential predictive and prognostic values of combined radiomics and volumetric analyses extracted from pretherapy 68Ga-PSMA PET-CT as well as clinical variables before 177Lu-PSMA therapy.

In total, data on radiomics approaches for the evaluation of the predictive or prognostic value of PSMA PET are still sparse. A small number of preliminary machine-learning radiomics studies evaluated the diagnostic performance of PET/CT imaging for predicting low vs. high lesion risk, biochemical recurrence, and overall patient risk using the machine learning approach in PC patients (30–32). Zamboglou et al. evaluated RF derived from [^68^Ga]-PSMA-11 PET/CT for intraprostatic tumor discrimination and non-invasive characterization of Gleason score and pelvic lymph node status (31). In uni- and multivariate analyses, QSZHGE was found to discriminate between GS 7 and GS ≥8 tumors, as well as between patients with pN1 disease and pN0 disease. It was demonstrated that RF could be used for non-invasive PCa diagnosis and characterization through [^68^Ga]-PSMA-11 PET/CT. As we know, in clinical practice, risk classification of primary PC is mainly based on the PSA level, Gleason score from biopsy samples, and tumor-nodes-metastasis staging. Papp et al. conducted a randomized prospective trial to investigate the diagnostic performance of PET/MRI using dual-tracer [18F]FMC and [68Ga]Ga-PSMA-11 for predicting low vs. high lesion risk as well as BCR and overall patient risk with machine learning in primary prostate cancer patients (30). In this study, radiomics and machine learning enhanced risk classification in primary prostate cancer patients without biopsy sampling (30). In another study, quantitative [18F]DCFPyL PET analysis using machine learning could predict lymph node involvement and high-risk pathological tumor characteristics in primary PCa patients (32). Therefore, a machine-learning radiomics algorithm was used to select 18F-Cho PET/CT imaging features to predict PCa disease progression using radiomics features analysis. An artificial intelligence model was found to be practical and had the potential to select a panel of 18F-Cho PET/CT features with noteworthy association with PCa patients’ outcomes (33). In our multicenter study, 33 mCRPC patients underwent several cycles of 177Lu-PSMA therapy of whom 22 cases (66%) showed BCR and 16 (48.5%) died during the follow-up time. We evaluated 17 baseline clinical factors and 65 extracted RFs from pretreatment 68Ga-PSMA PET-CT for prediction of response to 177Lu-PSMA therapy. According to the results, of 1 numerical and 16 parameters, age, number of treatment cycles, and administered dose had a significant correlation with BCR. In addition, among extracted radiomic features, GLCM entropy had a significant positive and GLZLM LZLGE had a significant negative correlation with BCR. HISTO energy showed a significant difference between BCR and no BCR patients but it had no significant correlation with BCR.

In prediction analysis of BCR, age and GLCM entropy showed the highest AUC with a sensitivity and specificity of 82%-73% and 64%-82%, respectively. In addition, GLZLM LZLGE showed the highest AUC with a sensitivity of 100% and specificity of 50% for the prediction of no BCR. According to SVM, an AUC ROC of 64-76% was achieved for the prediction of response to therapy using the selected parameters including age, GLCM entropy, and GLZLM LZLGE among which the best result was achieved with GLCM entropy alone. In other words, GLCM entropy alone showed the highest predictive performance in treatment planning. In line with previous studies, SUV parameters as conventional PET parameters showed no significant predictive value for BCR (27). Moreover, similar to previous studies, GLCM entropy as a heterogeneity parameter could be used as a predictive index for BCR (7). Moazemi et al. (34) evaluated the predictive values of RFs extracted from pretreatment 68Ga-PSMA PET-CT and clinical parameters for response to 177Lu-PSMA therapy. They found that SUV_min_ (first-ordered) and correlation (textural) among PET extracted features, Min (first-ordered), Busyness (textural), and Coarseness (textural) among CT features, and clinical parameters had the best correlation with PSA response. Their model had an AUC of 80%, the sensitivity of 75%, and specificity of 75% for treatment response prediction using the SVM classifier. In another study, the predictive value of RFs extracted from pretreatment 68Ga-PET-MRI on BCR was evaluated. The results showed that three PET-derived (interquartile range PET, mean PET, median PET one T2-derived (interquartile range T2) and four T1-post-GD-derived parameters (interquartile range T1GD, Entropy T1 GD, mean absolute deviation T1GD, cluster tendency T1GD, Imc2 T1GD, SumEntropy T1GD) differentiated well between BCR and no BCR patients (35).

We found no significant relationship between BCR and D_max_, total PSMA-TV, PSMA-TV_max_, PSMA-TV_mean_, TL-PSMA_max_, TL-PSMA_mean_, total TL-PSMA, and TLR. This finding was in contrast to the results of a study by A. Aksu et al. that found that D_max_ was a prognostic parameter for the prediction of early BCR after 177Lu-PSMA therapy (36). In addition, Fadi Khreish et al. found that 68Ga-PSMA PET/CT-derived TLR could be used as a predictor for PFS after 177Lu-PSMA therapy (37).

Tumor heterogeneity can be considered an important factor affecting response to therapy, which refers to the presence of distinct populations of cells exhibiting different phenotypes and genotypes either within the primary tumor and its metastases or between tumors of the same histopathological subtype (intra- and inter-tumor heterogeneity, respectively) (38). Several procedures have been introduced for the evaluation of tumor heterogeneity including *in vitro* molecular pathology, serum-based biomarkers, liquid biopsy, pharmacogenomic-based markers, and molecular imaging-based biomarkers. Conventional biomarkers used in the clinical setting, such as the serum PSA level, do not provide adequate results to assess the effectiveness of treatment in tumors with mixed cellular patterns. Therefore, we choose to focus on ‘response predictors’ based on the various modalities in use to assess treatment response. As for the evaluation of tumor heterogeneity, imaging has different advantages over conventional methods like random biopsy, especially in mCRPC patients presenting with several metastases. First, imaging offers a full 3D volume assessment of the tumor as well as different metastases originating from the same organ or other organs. Second, it allows for a longitudinal study. Finally, imaging makes it possible to assess heterogeneity between patients with similar tumors (interpatient heterogeneity), between different tumors within each patient (intertumor heterogeneity), and within the tissue itself (intratumor heterogeneity) (28). According to the results of the present, patients with higher entropy resulting in more heterogenous lesions were more responsive to 177Lu-PSMA therapy. This finding was consistent with the results of a study by Khurshid et al. that found that heterogeneity parameters from pretreatment 68Ga-PSMA PET-CT including homogeneity and entropy had significant predictive values for response to 177Lu-PSMA therapy (39).

In the present study, patients with BCR lived significantly longer compared to no BCR patients, which is in line with a previous study (35); moreover, neither RFs nor clinical parameters had significant predictive values for OS. Moazemi et al. (40) assessed the predictive values of RFs extracted from pretreatment 68Ga-PSMA PET-CT and baseline clinical parameters for OS in patients undergoing 177Lu-PSMA therapy. They found that SUV_min_, SUV_mean_, and kurtosis as RFs and baseline values of hemoglobin and C-reactive protein (CRP), and Eastern Cooperative Oncology Group (ECOG) performance status scale as clinical parameters had a significant impact on OS. Their results were in contrast to other studies that found that conventional PET parameters such as SUV parameters had no predictive value for OS (41–43).

It is important to note that this study had some limitations, especially its retrospective design and small sample size with few data entry points. Furthermore, the progression free survival was not available for all the patients. In addition, malignant lesions were determined by a nuclear medicine specialist without histopathological information. Though we just had 33 patients, we assessed 2517 pathological hotspots in total, so it could demonstrate statistical significance in this study; however, further prospective studies need to be carried out to verify these findings.

## Conclusion

This exploratory study showed that PSMA uptake heterogeneity in mCRPC acquired by radiomics analysis from pre-treatment 68Ga-PSMA PET-CT images may be associated with a PSA response. However, further well-designed studies are needed to validate if prostate cancer PSMA heterogeneity quantification could offer appreciated predictive and prognostic values for treatment planning in these patients.

## Data availability statement

The original contributions presented in the study are included in the article/**Supplementary Material**. Further inquiries can be directed to the corresponding author.

## Ethics statement

Ethical approval was not provided for this study on human participants because We used available data and this is a retrospective study. The patients/participants provided their written informed consent to participate in this study.

## Author contributions

MA: Data collection, writing, study design. RM-F: Data collection, data analyzing. EJ: Data collection, Data analyzing, writing. AK: Study design, data analyzing. GD: data collection. MM: data collection. ZA: data collection. RS: data collection. HD: data collection. NJ: writing. BM: statistical analyzing. VP: writing, study design, supervision. All authors contributed to the article and approved the submitted version.
