# Supplementary material for: Predictive and prognostic potential of pretreatment 68Ga-PSMA PET tumor heterogeneity index in patients with metastatic castration-resistant prostate cancer treated with 177Lu-PSMA
Source: Front Oncol. 2022 Dec 8;12:1066926. doi: 10.3389/fonc.2022.1066926 (PMC9773988; doi:10.3389/fonc.2022.1066926)
Supplement: Supplementary file 1 [file Presentation_1.pptx]

## Slide 1
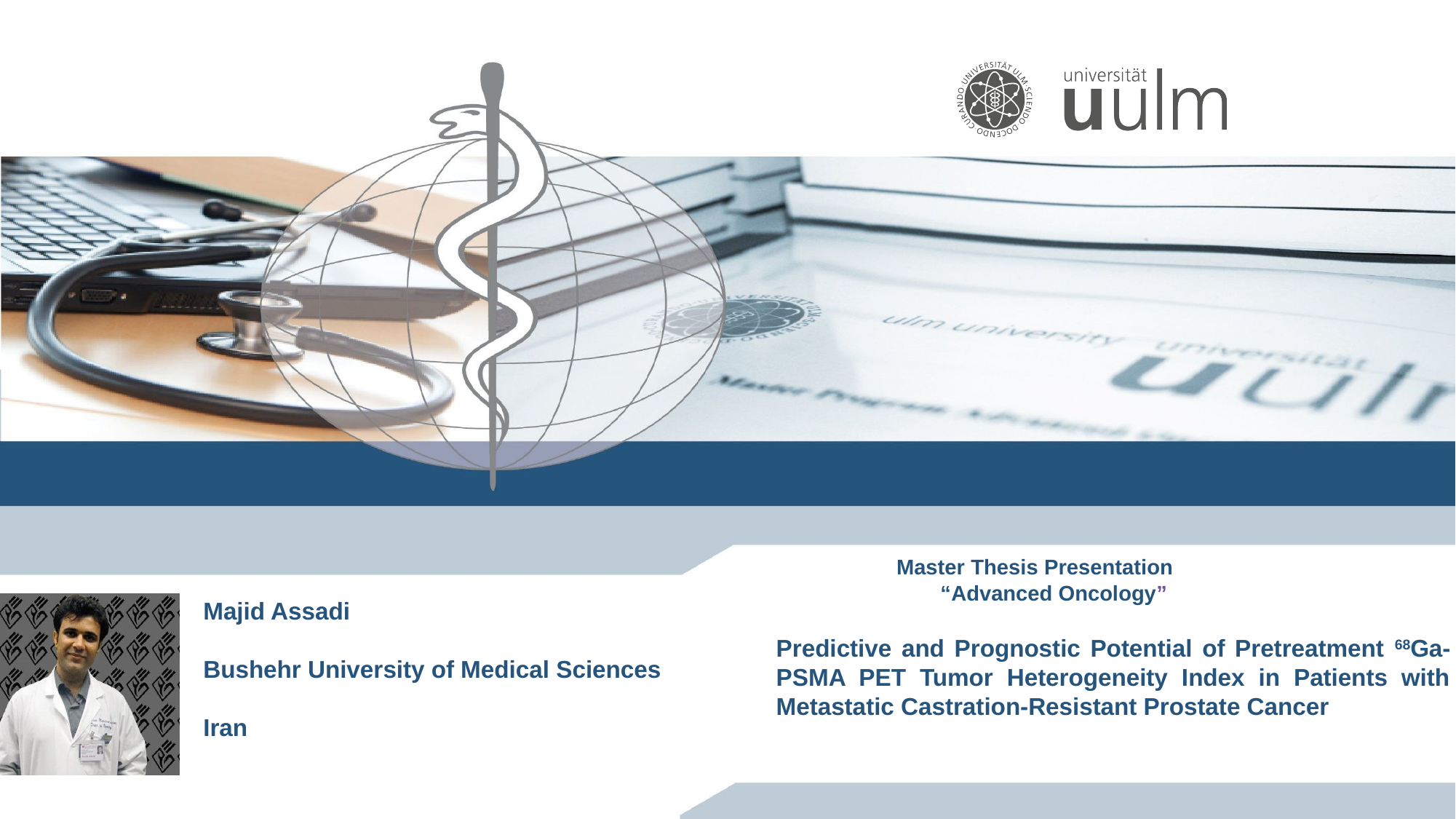

Master Thesis Presentation
“Advanced Oncology”
Majid Assadi
Bushehr University of Medical Sciences
Iran
Predictive and Prognostic Potential of Pretreatment 68Ga-PSMA PET Tumor Heterogeneity Index in Patients with Metastatic Castration-Resistant Prostate Cancer

## Slide 2
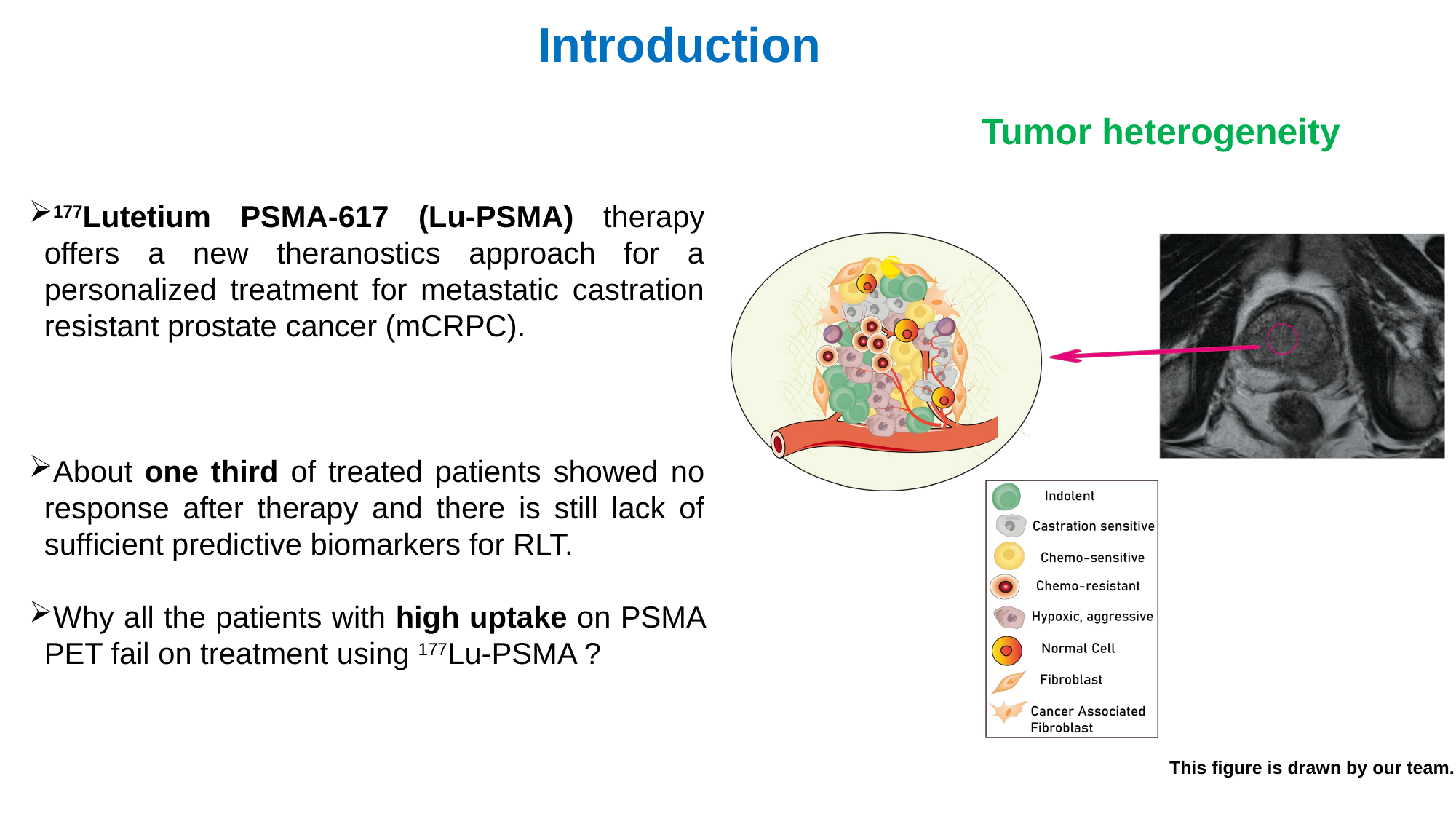

# Introduction
 Tumor heterogeneity
177Lutetium PSMA-617 (Lu-PSMA) therapy offers a new theranostics approach for a personalized treatment for metastatic castration resistant prostate cancer (mCRPC).
About one third of treated patients showed no response after therapy and there is still lack of sufficient predictive biomarkers for RLT.
Why all the patients with high uptake on PSMA PET fail on treatment using 177Lu-PSMA ?
This figure is drawn by our team.

## Slide 3
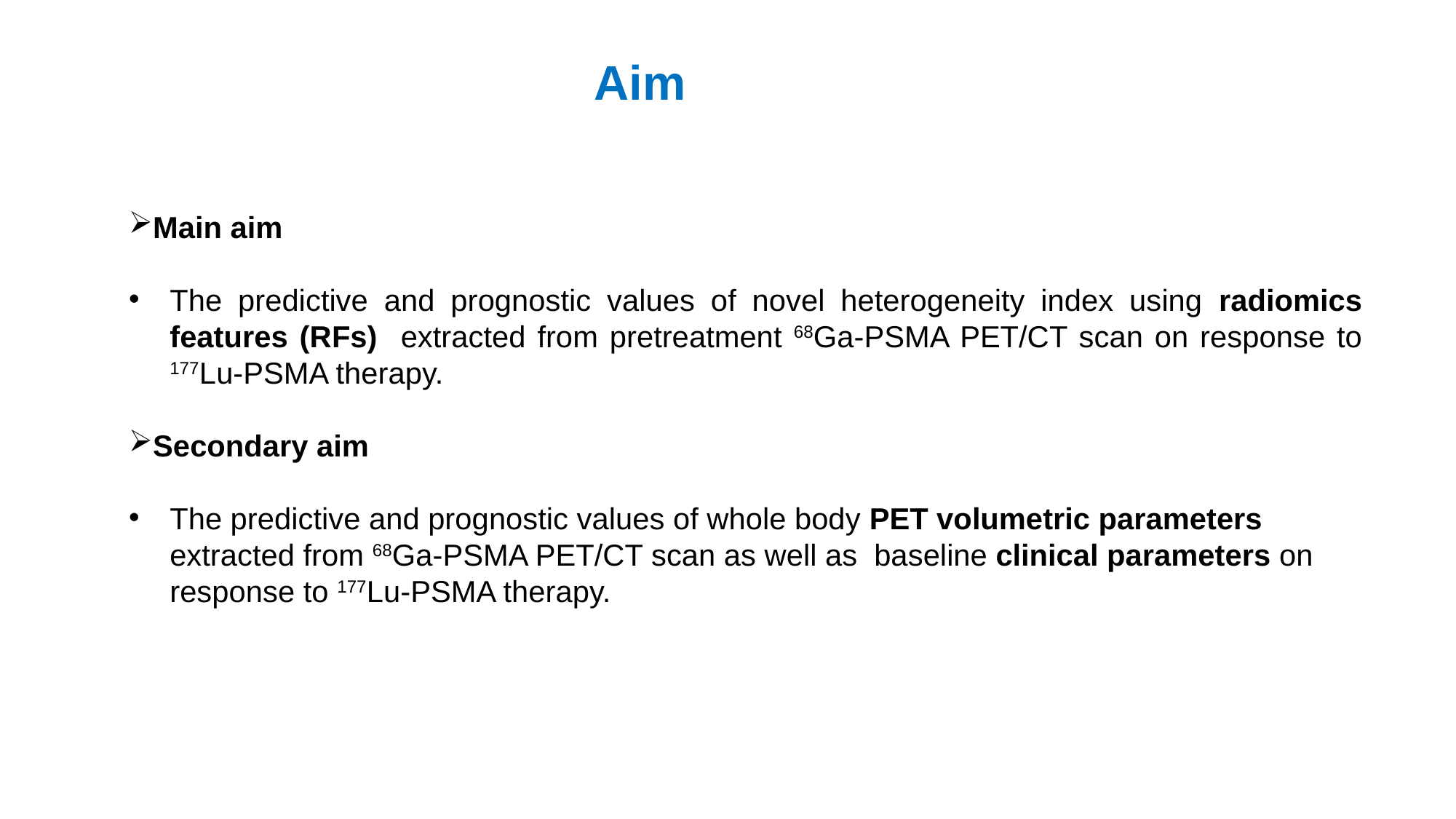

# Aim
Main aim
The predictive and prognostic values of novel heterogeneity index using radiomics features (RFs) extracted from pretreatment 68Ga-PSMA PET/CT scan on response to 177Lu-PSMA therapy.
Secondary aim
The predictive and prognostic values of whole body PET volumetric parameters extracted from 68Ga-PSMA PET/CT scan as well as baseline clinical parameters on response to 177Lu-PSMA therapy.

## Slide 4
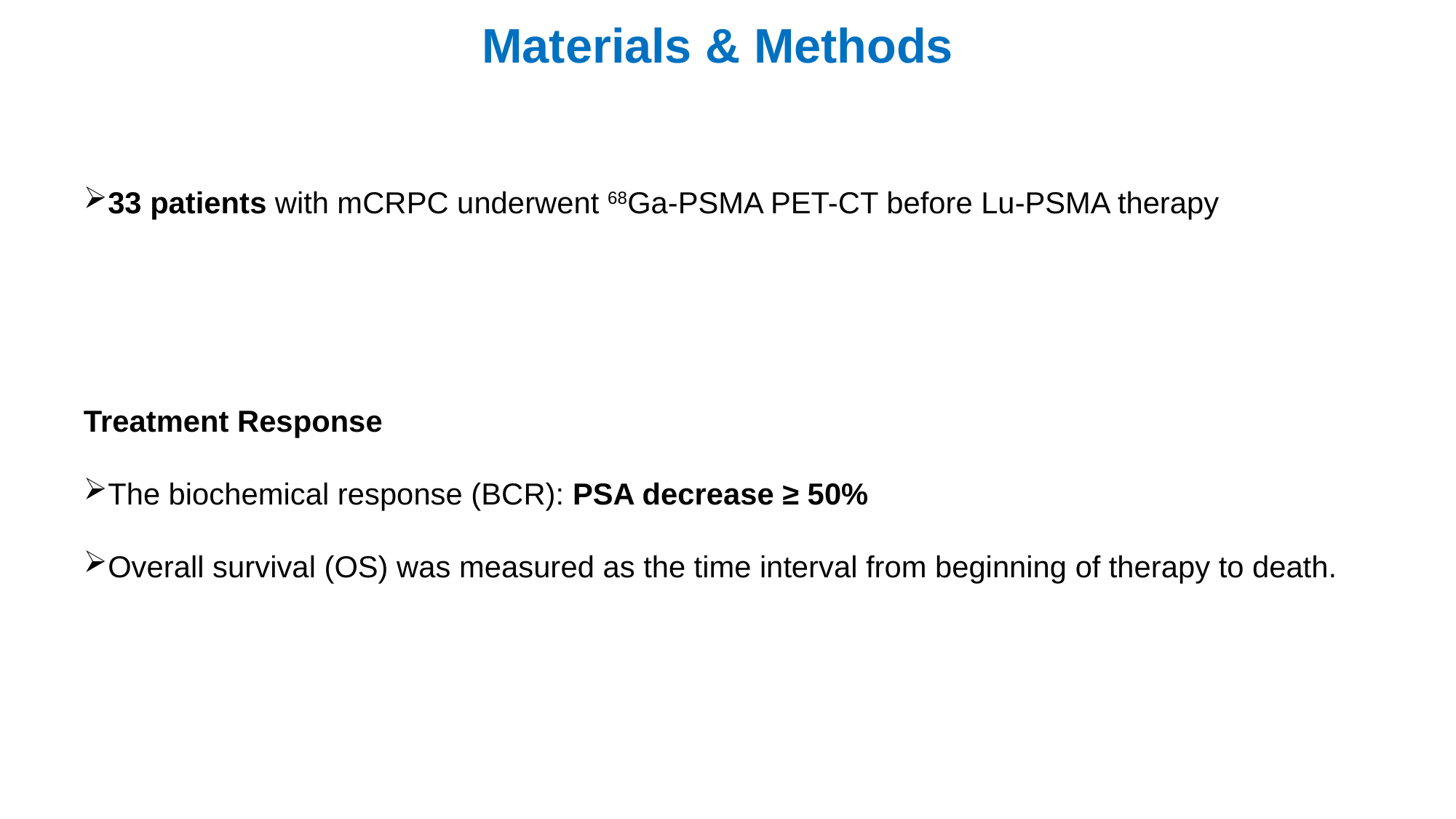

# Materials & Methods
33 patients with mCRPC underwent 68Ga-PSMA PET-CT before Lu-PSMA therapy
Treatment Response
The biochemical response (BCR): PSA decrease ≥ 50%
Overall survival (OS) was measured as the time interval from beginning of therapy to death.

## Slide 5
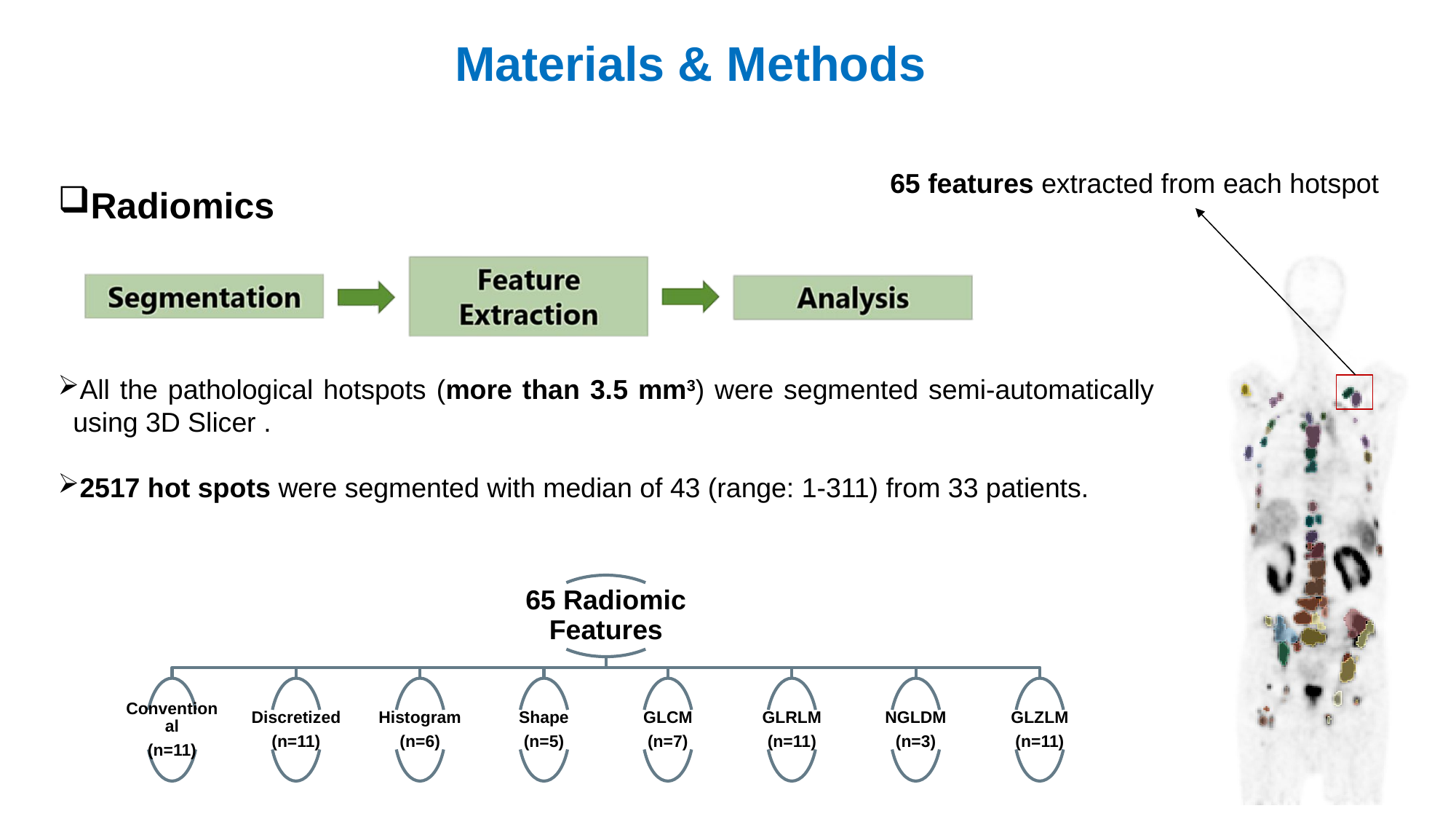

# Materials & Methods
65 features extracted from each hotspot
Radiomics
All the pathological hotspots (more than 3.5 mm3) were segmented semi-automatically using 3D Slicer .
2517 hot spots were segmented with median of 43 (range: 1-311) from 33 patients.

## Slide 6
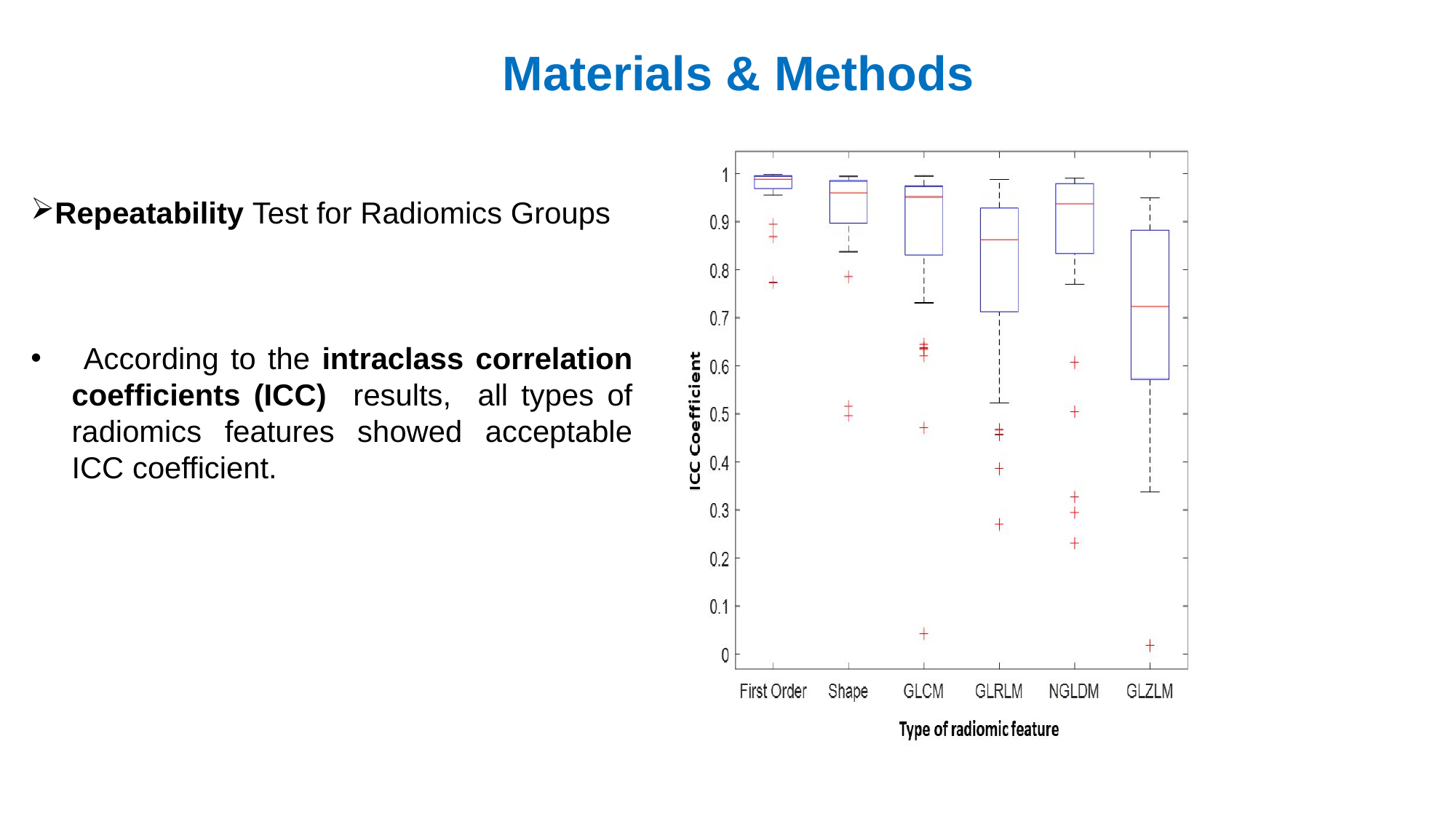

Materials & Methods
Repeatability Test for Radiomics Groups
 According to the intraclass correlation coefficients (ICC) results, all types of radiomics features showed acceptable ICC coefficient.

## Slide 7
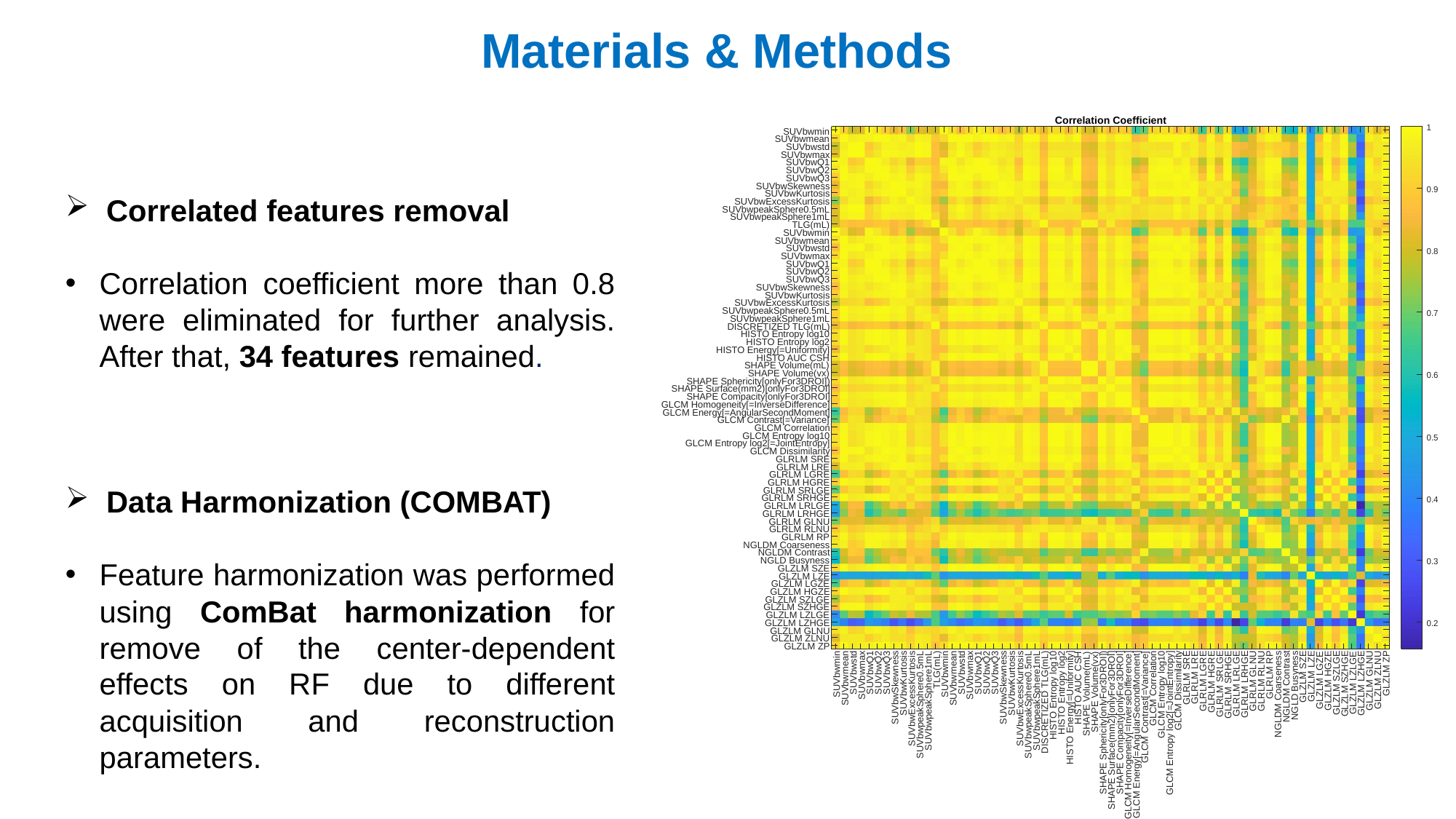

Materials & Methods
Correlated features removal
Correlation coefficient more than 0.8 were eliminated for further analysis. After that, 34 features remained.
Data Harmonization (COMBAT)
Feature harmonization was performed using ComBat harmonization for remove of the center-dependent effects on RF due to different acquisition and reconstruction parameters.

## Slide 8
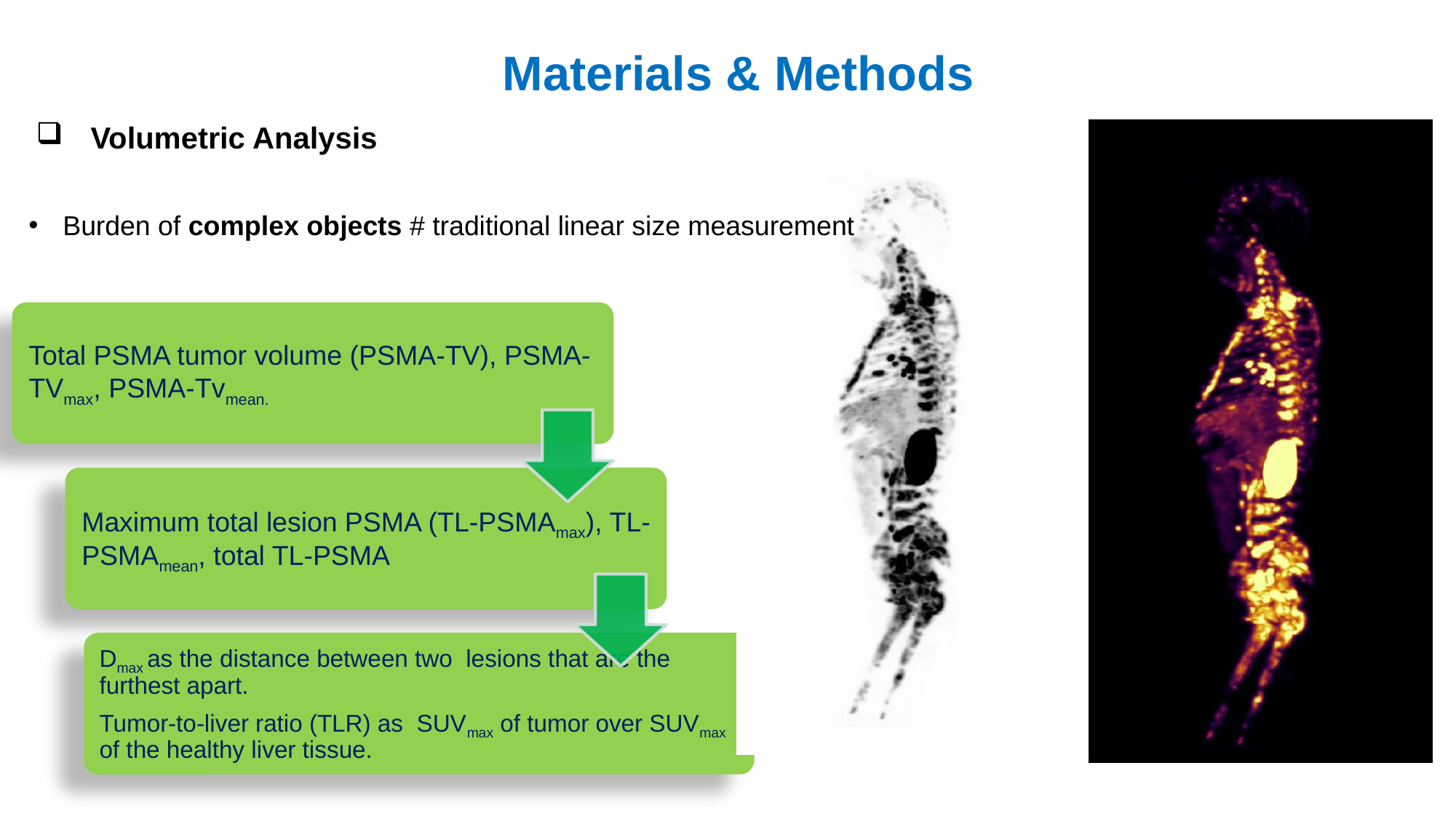

Materials & Methods
# Volumetric Analysis
Burden of complex objects # traditional linear size measurement

## Slide 9
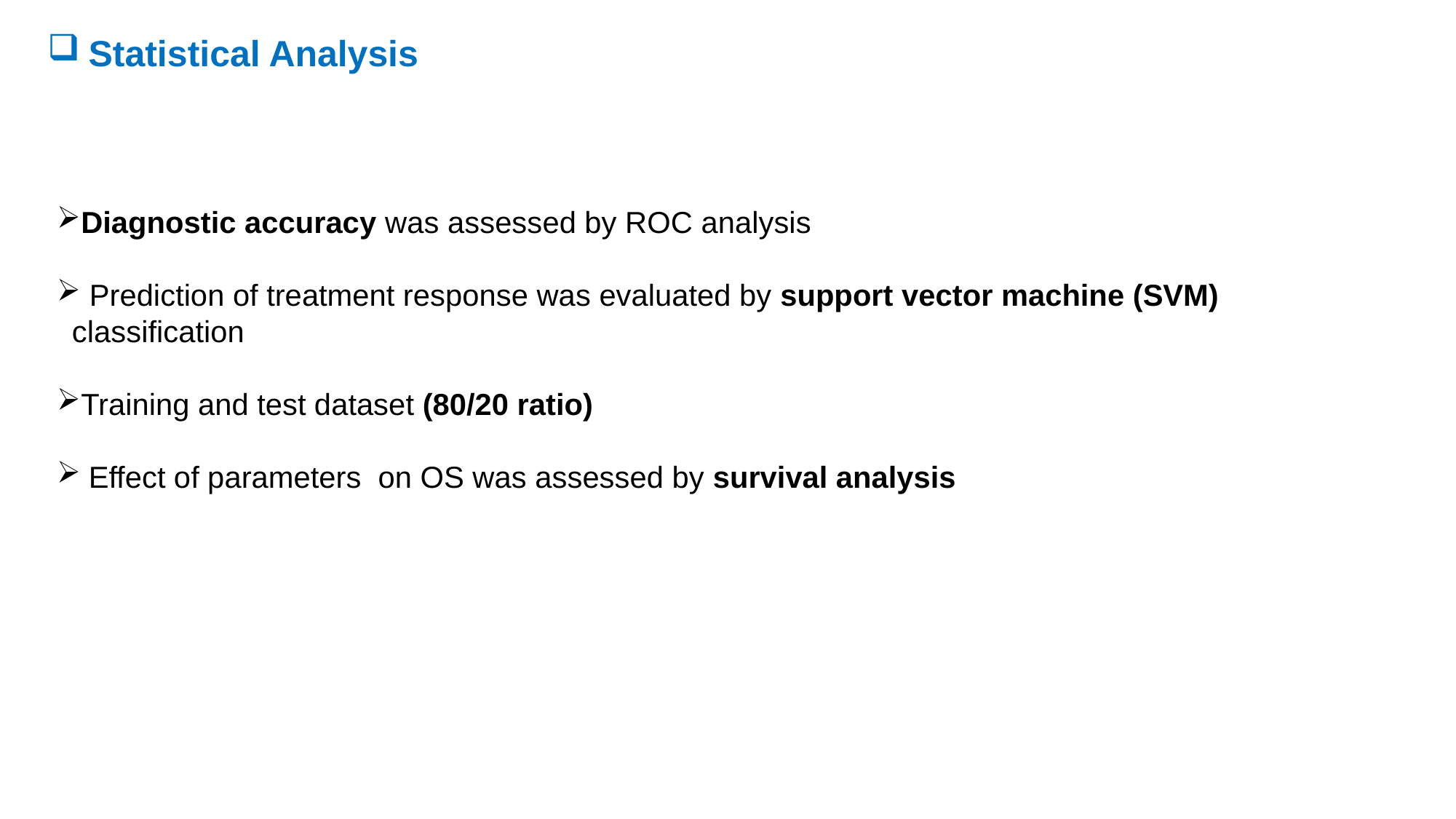

# Statistical Analysis
Diagnostic accuracy was assessed by ROC analysis
 Prediction of treatment response was evaluated by support vector machine (SVM) classification
Training and test dataset (80/20 ratio)
﻿ Effect of parameters on OS was assessed by survival analysis

## Slide 10
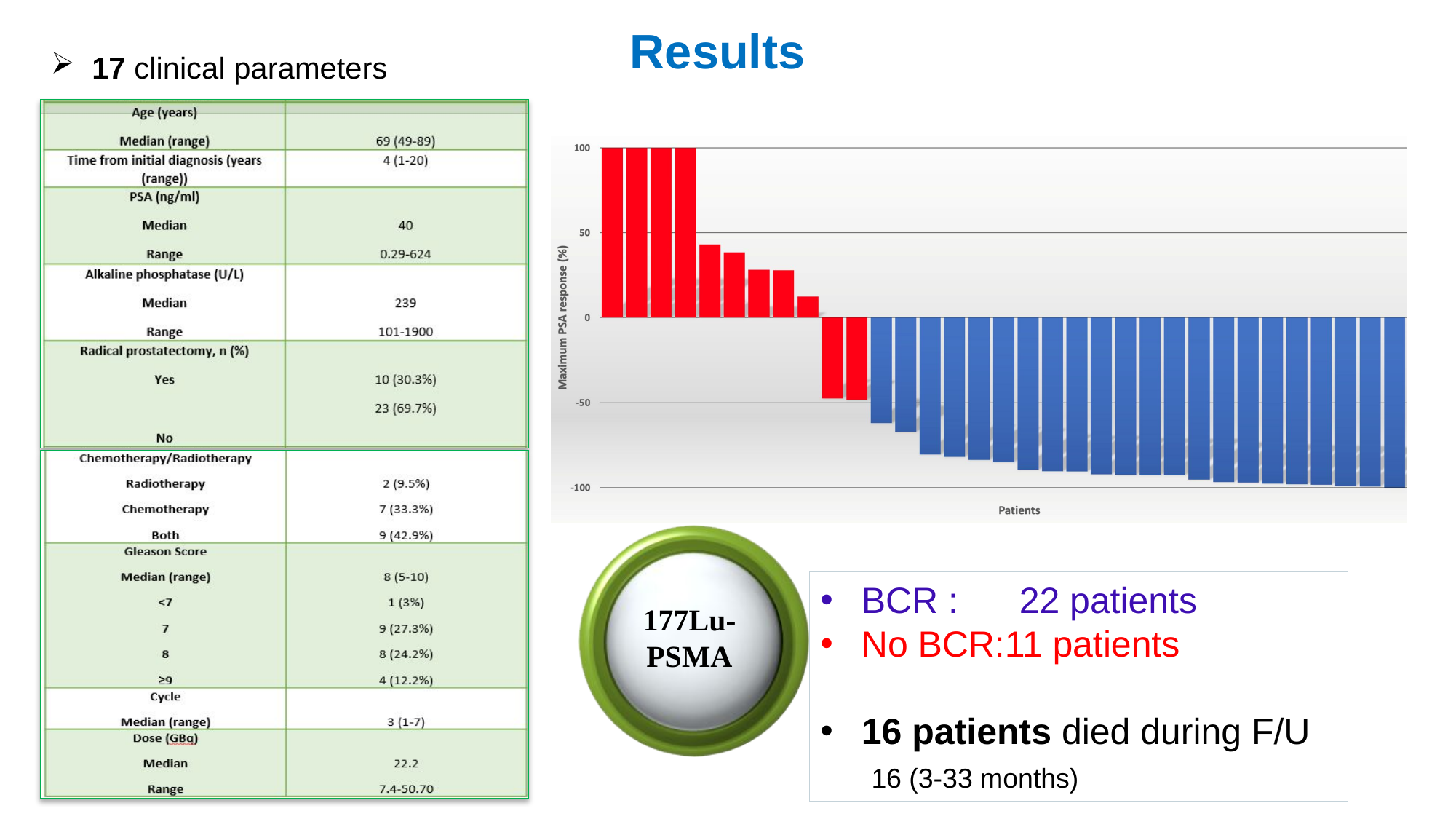

Results
17 clinical parameters
BCR : 22 patients
No BCR:11 patients
16 patients died during F/U
 16 (3-33 months)
177Lu-PSMA

## Slide 11
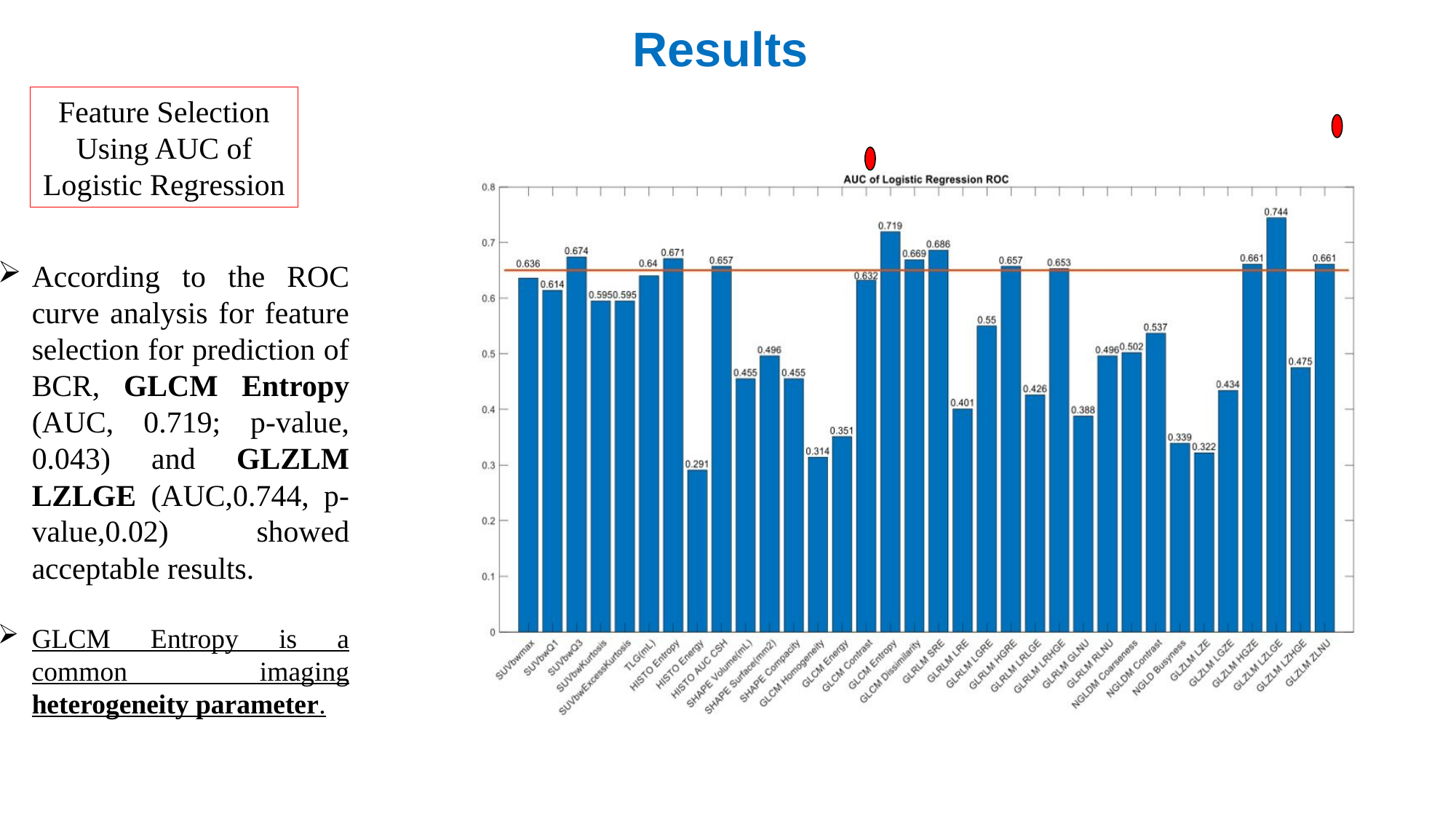

Results
Feature Selection Using AUC of Logistic Regression
According to the ROC curve analysis for feature selection for prediction of BCR, GLCM Entropy (AUC, 0.719; p-value, 0.043) and GLZLM LZLGE (AUC,0.744, p-value,0.02) showed acceptable results.
GLCM Entropy is a common imaging heterogeneity parameter.

## Slide 12
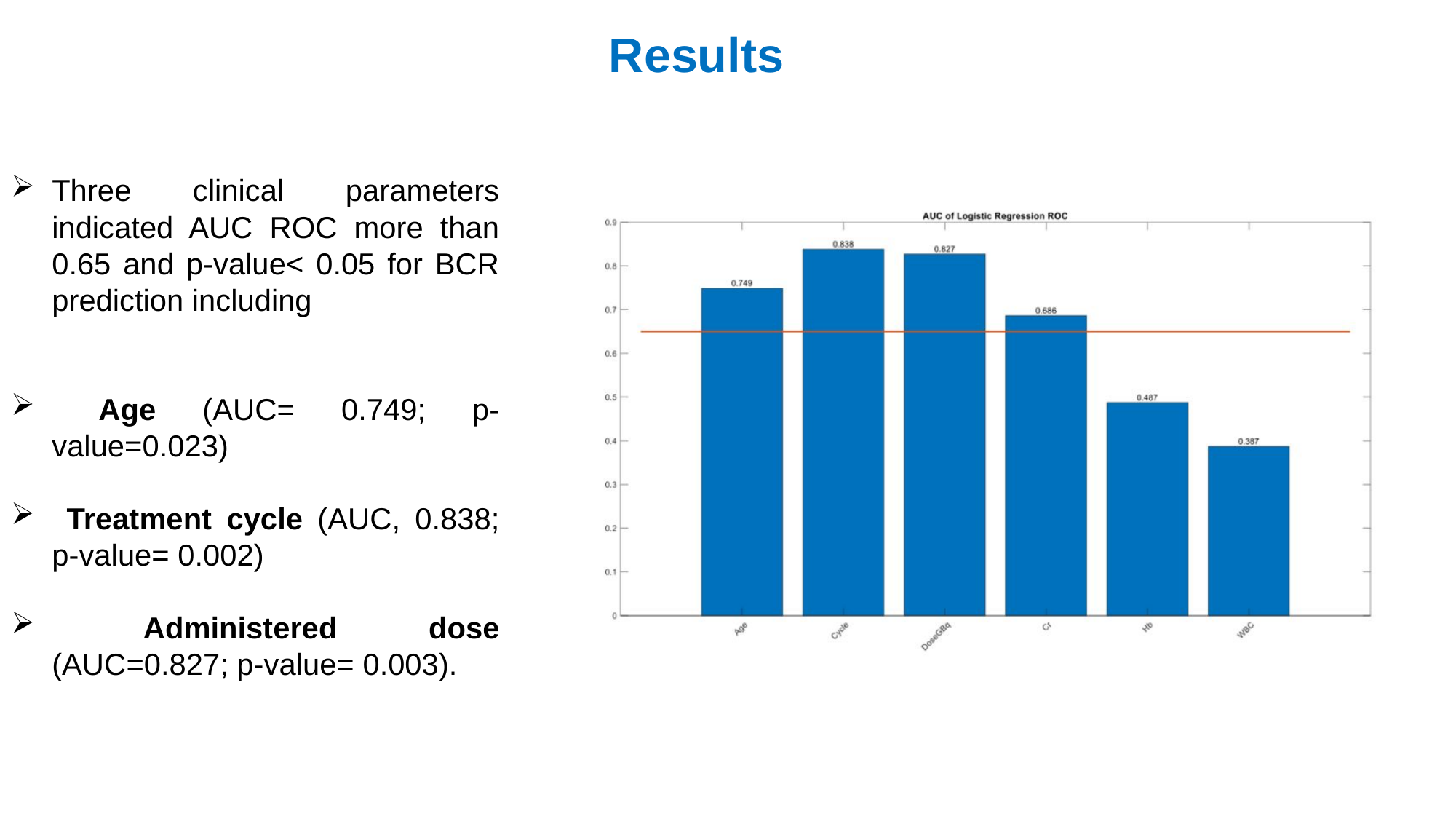

Results
Three clinical parameters indicated AUC ROC more than 0.65 and p-value< 0.05 for BCR prediction including
 Age (AUC= 0.749; p-value=0.023)
 Treatment cycle (AUC, 0.838; p-value= 0.002)
 Administered dose (AUC=0.827; p-value= 0.003).

## Slide 13
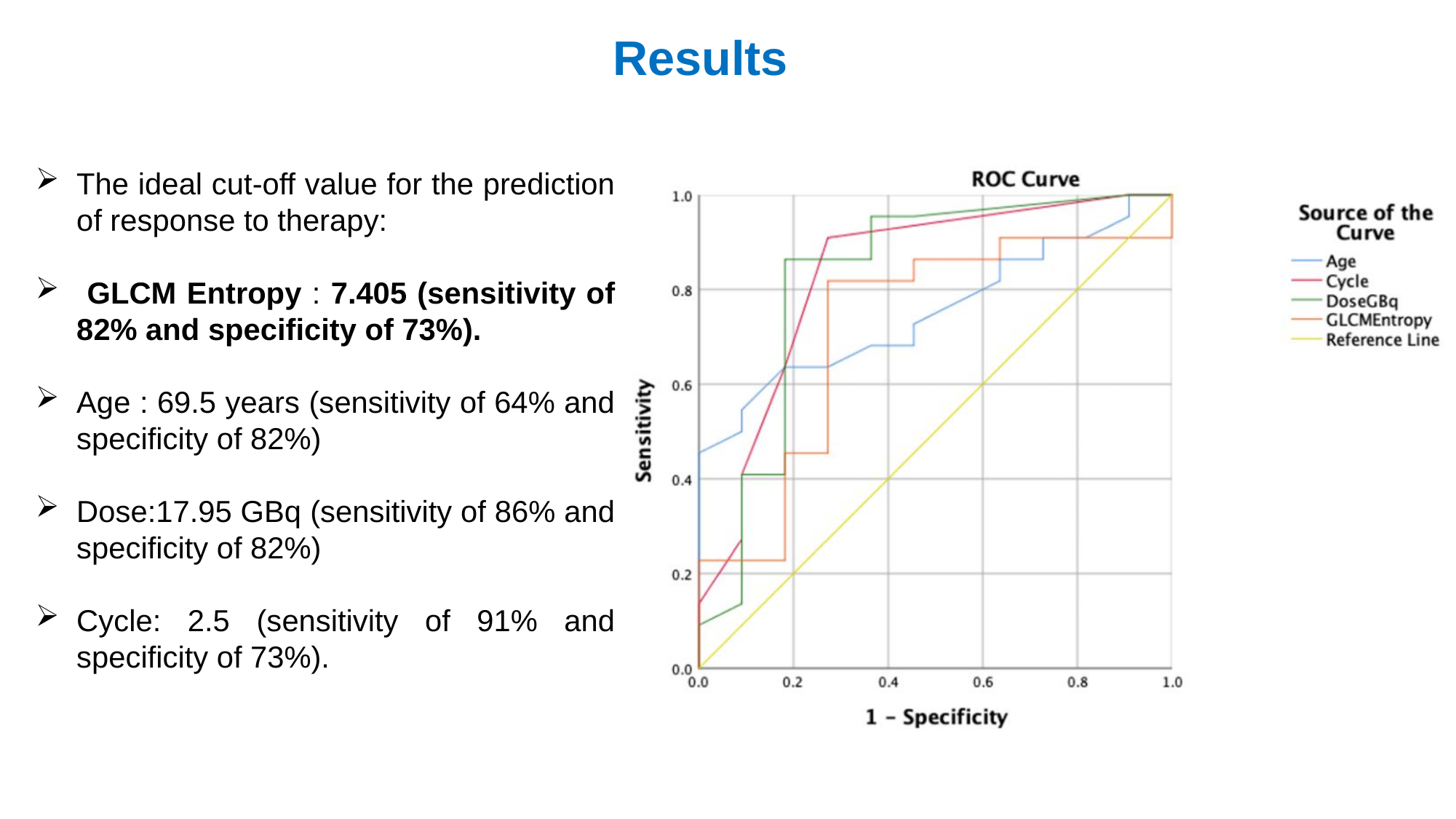

Results
The ideal cut-off value for the prediction of response to therapy:
 GLCM Entropy : 7.405 (sensitivity of 82% and specificity of 73%).
Age : 69.5 years (sensitivity of 64% and specificity of 82%)
Dose:17.95 GBq (sensitivity of 86% and specificity of 82%)
Cycle: 2.5 (sensitivity of 91% and specificity of 73%).

## Slide 14
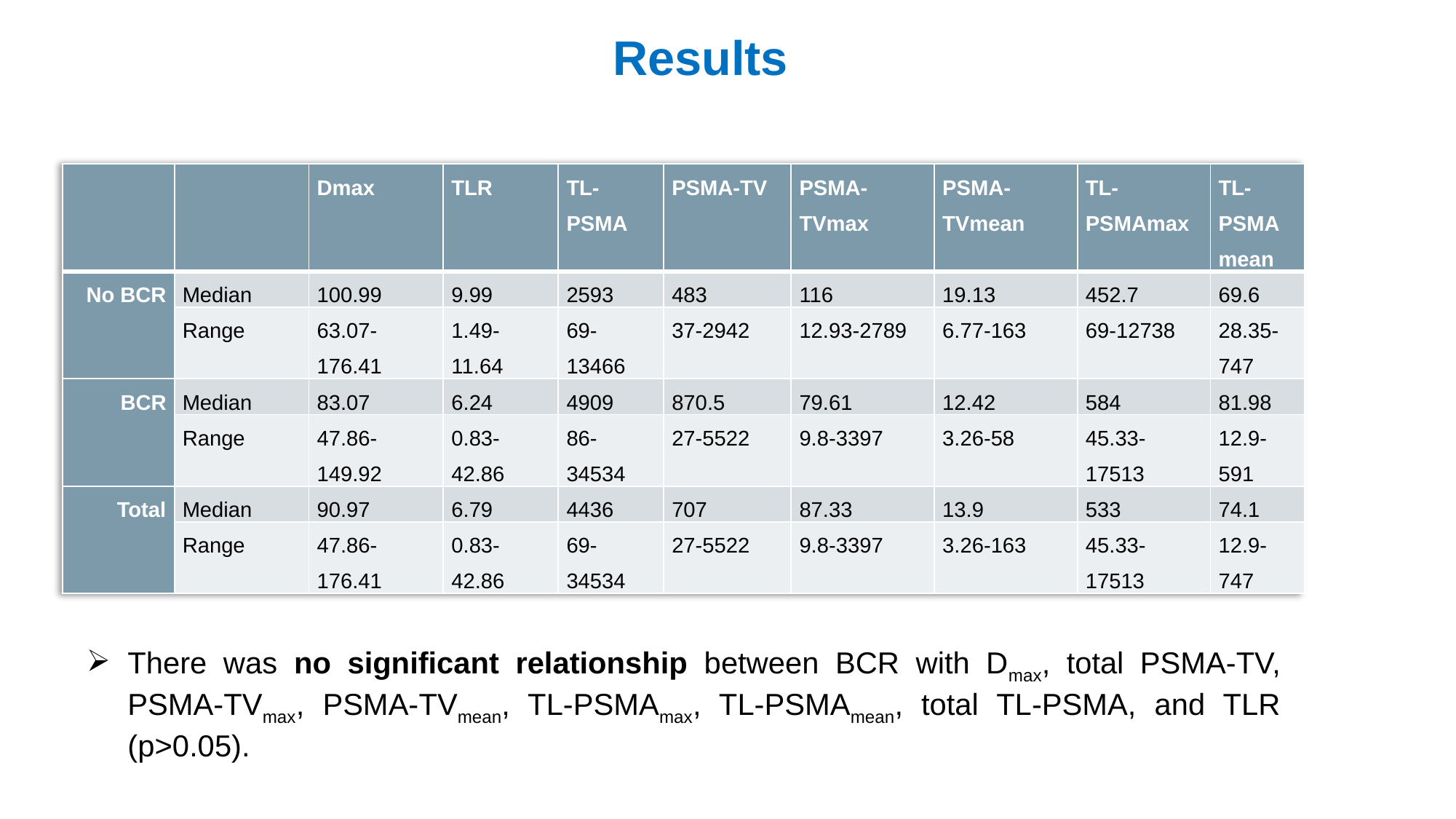

Results
| | | Dmax | TLR | TL-PSMA | PSMA-TV | PSMA-TVmax | PSMA-TVmean | TL-PSMAmax | TL-PSMAmean |
| --- | --- | --- | --- | --- | --- | --- | --- | --- | --- |
| No BCR | Median | 100.99 | 9.99 | 2593 | 483 | 116 | 19.13 | 452.7 | 69.6 |
| | Range | 63.07-176.41 | 1.49-11.64 | 69-13466 | 37-2942 | 12.93-2789 | 6.77-163 | 69-12738 | 28.35-747 |
| BCR | Median | 83.07 | 6.24 | 4909 | 870.5 | 79.61 | 12.42 | 584 | 81.98 |
| | Range | 47.86-149.92 | 0.83-42.86 | 86-34534 | 27-5522 | 9.8-3397 | 3.26-58 | 45.33-17513 | 12.9-591 |
| Total | Median | 90.97 | 6.79 | 4436 | 707 | 87.33 | 13.9 | 533 | 74.1 |
| | Range | 47.86-176.41 | 0.83-42.86 | 69-34534 | 27-5522 | 9.8-3397 | 3.26-163 | 45.33-17513 | 12.9-747 |
There was no significant relationship between BCR with Dmax, total PSMA-TV, PSMA-TVmax, PSMA-TVmean, TL-PSMAmax, TL-PSMAmean, total TL-PSMA, and TLR (p>0.05).

## Slide 15
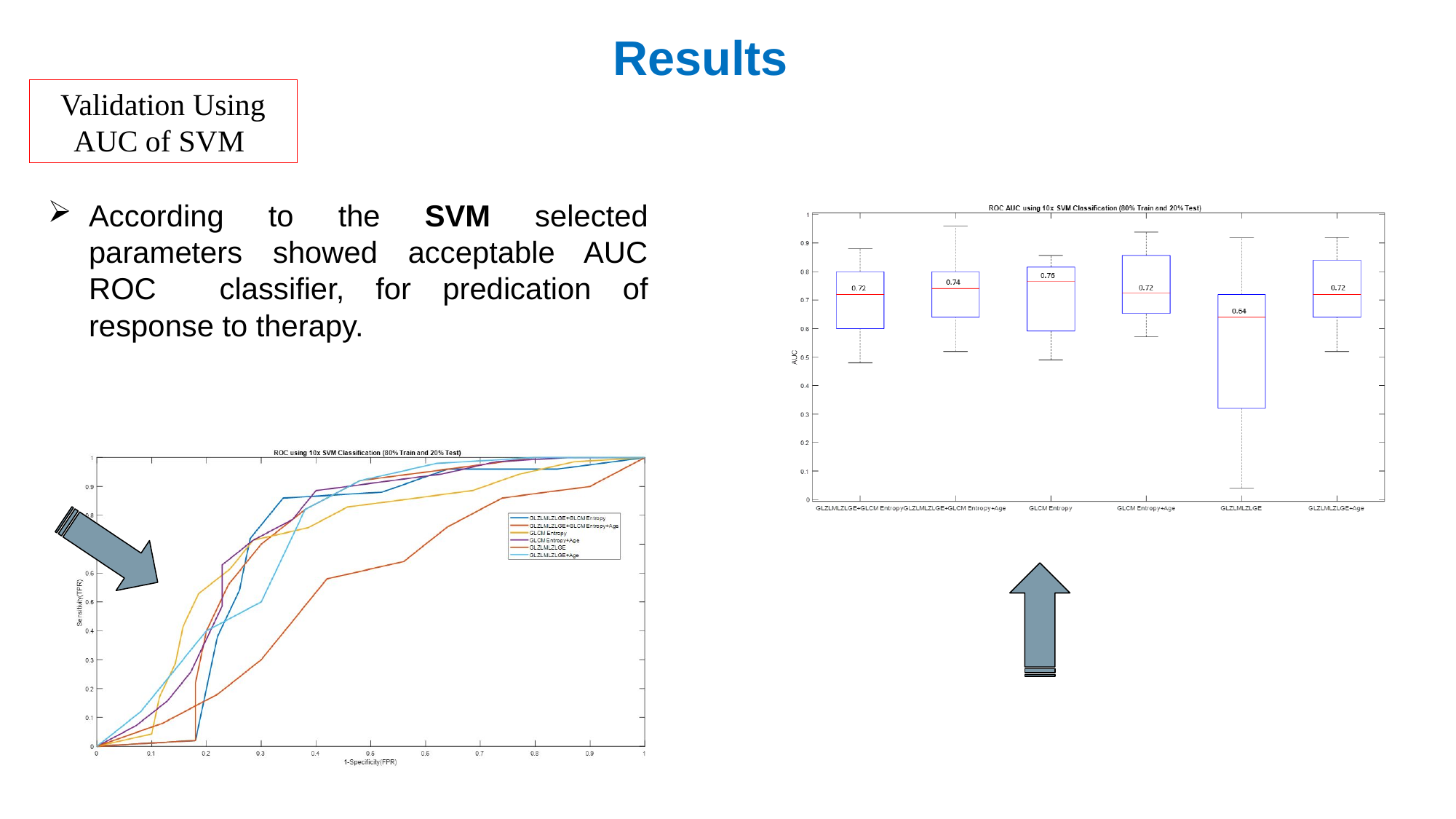

Results
Validation Using AUC of SVM
According to the SVM selected parameters showed acceptable AUC ROC classifier, for predication of response to therapy.

## Slide 16
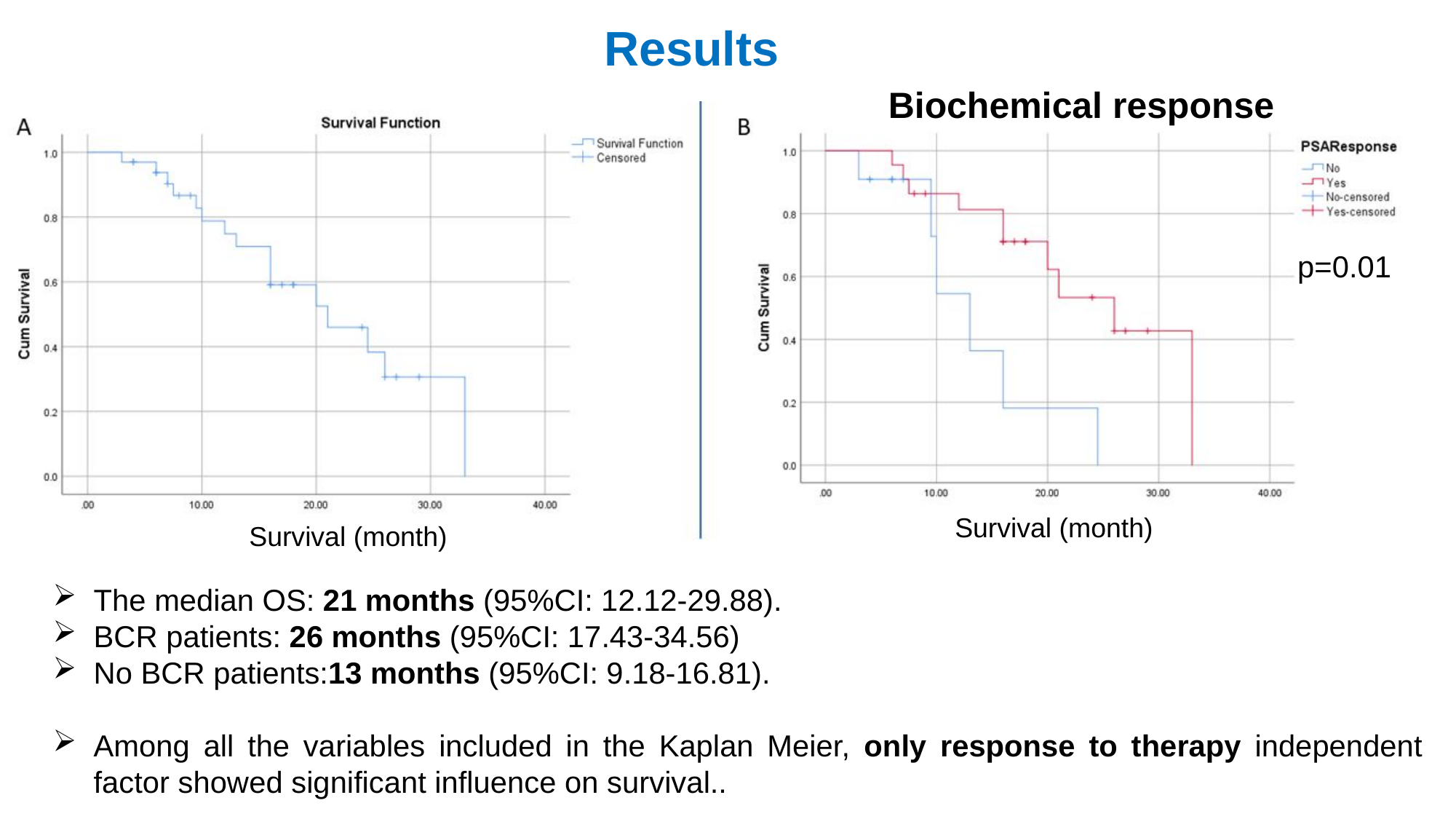

Results
Biochemical response
p=0.01
Survival (month)
Survival (month)
The median OS: 21 months (95%CI: 12.12-29.88).
BCR patients: 26 months (95%CI: 17.43-34.56)
No BCR patients:13 months (95%CI: 9.18-16.81).
Among all the variables included in the Kaplan Meier, only response to therapy independent factor showed significant influence on survival..

## Slide 17
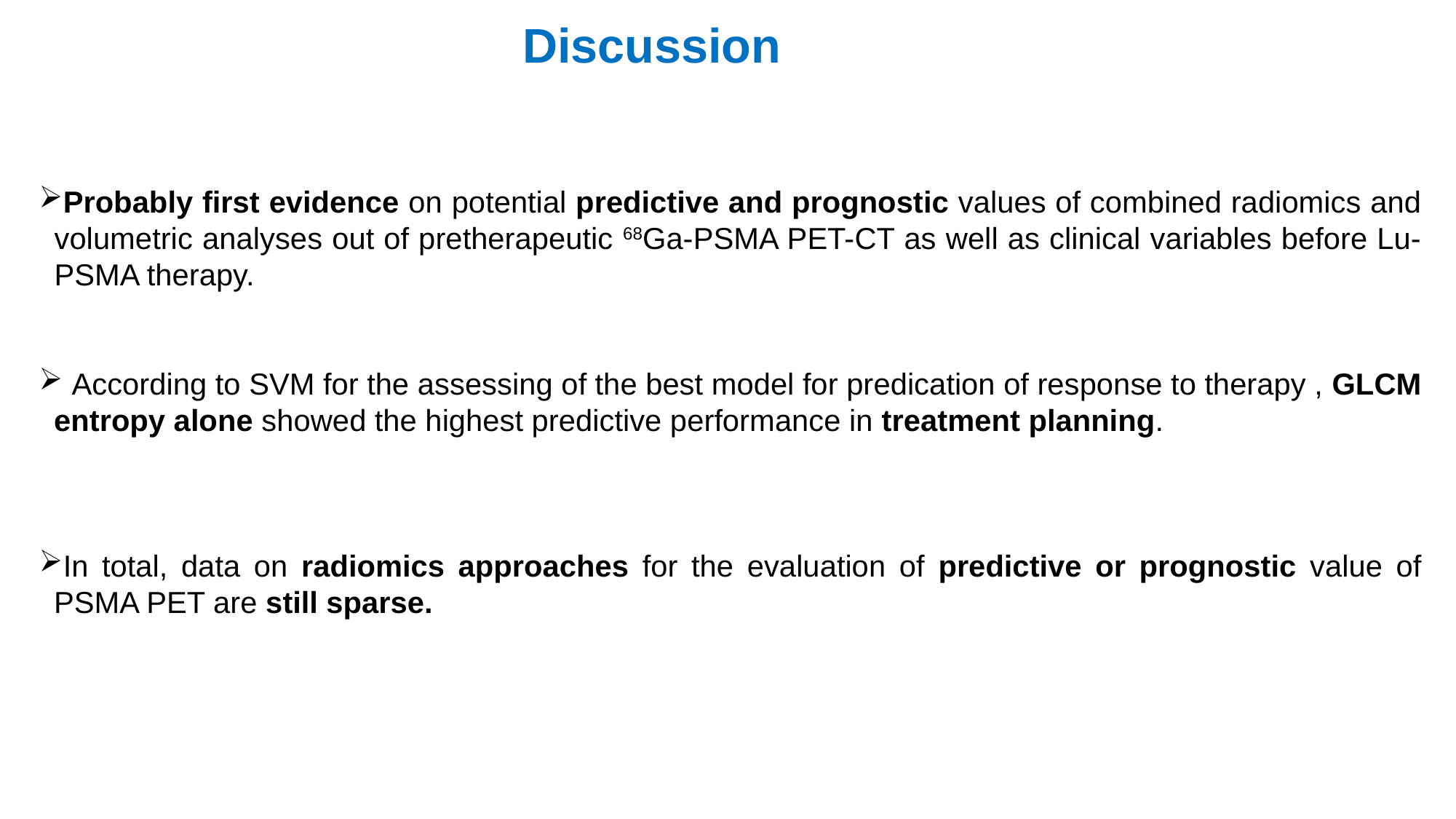

Discussion
Probably first evidence on potential predictive and prognostic values of combined radiomics and volumetric analyses out of pretherapeutic 68Ga-PSMA PET-CT as well as clinical variables before Lu-PSMA therapy.
 According to SVM for the assessing of the best model for predication of response to therapy , GLCM entropy alone showed the highest predictive performance in treatment planning.
In total, data on radiomics approaches for the evaluation of predictive or prognostic value of PSMA PET are still sparse.

## Slide 18
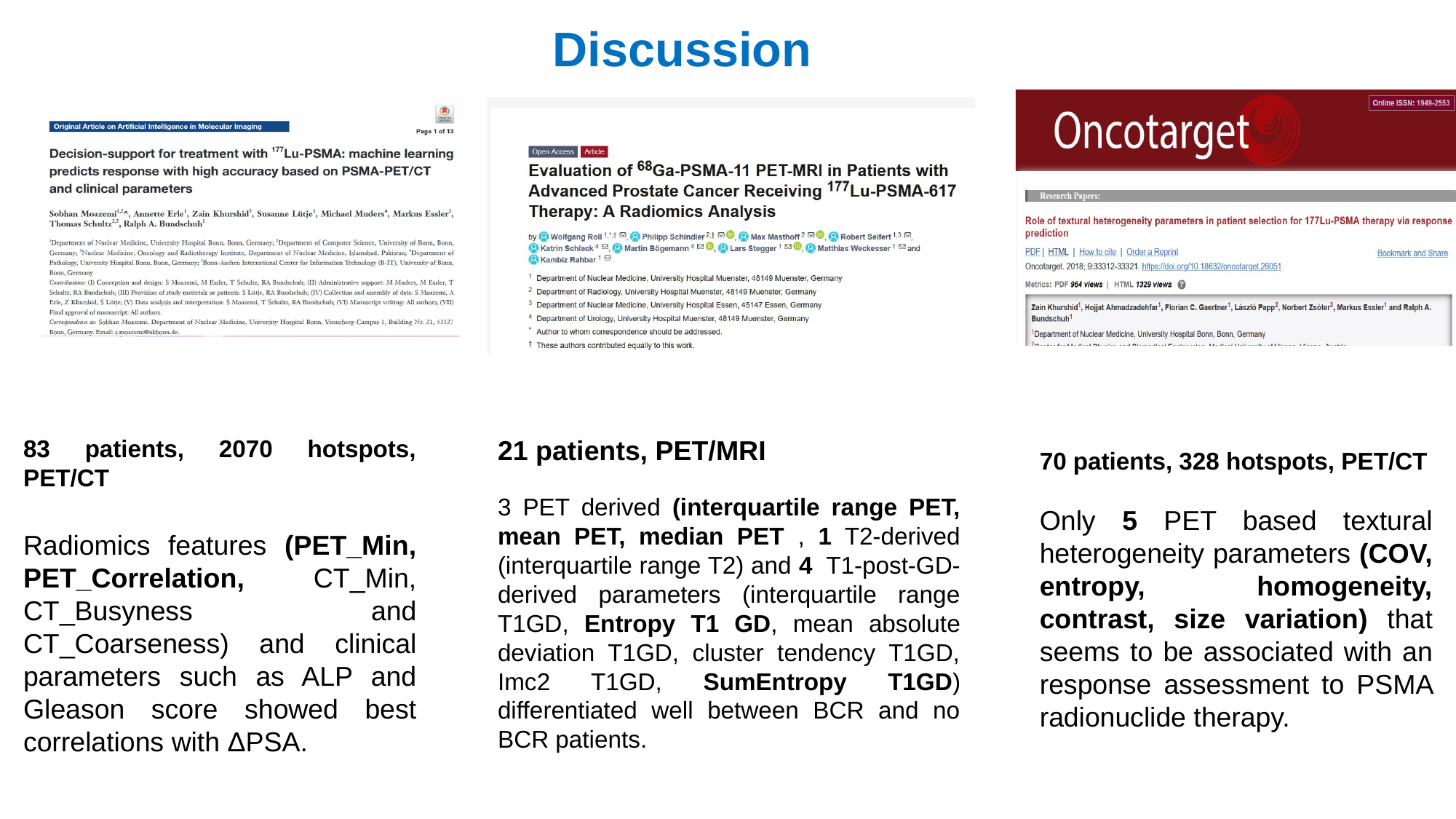

Discussion
83 patients, 2070 hotspots, PET/CT
Radiomics features (PET_Min, PET_Correlation, CT_Min, CT_Busyness and CT_Coarseness) and clinical parameters such as ALP and Gleason score showed best correlations with ΔPSA.
21 patients, PET/MRI
3 PET derived (interquartile range PET, mean PET, median PET , 1 T2-derived (interquartile range T2) and 4 T1-post-GD-derived parameters (interquartile range T1GD, Entropy T1 GD, mean absolute deviation T1GD, cluster tendency T1GD, Imc2 T1GD, SumEntropy T1GD) differentiated well between BCR and no BCR patients.
70 patients, 328 hotspots, PET/CT
Only 5 PET based textural heterogeneity parameters (COV, entropy, homogeneity, contrast, size variation) that seems to be associated with an response assessment to PSMA radionuclide therapy.

## Slide 19
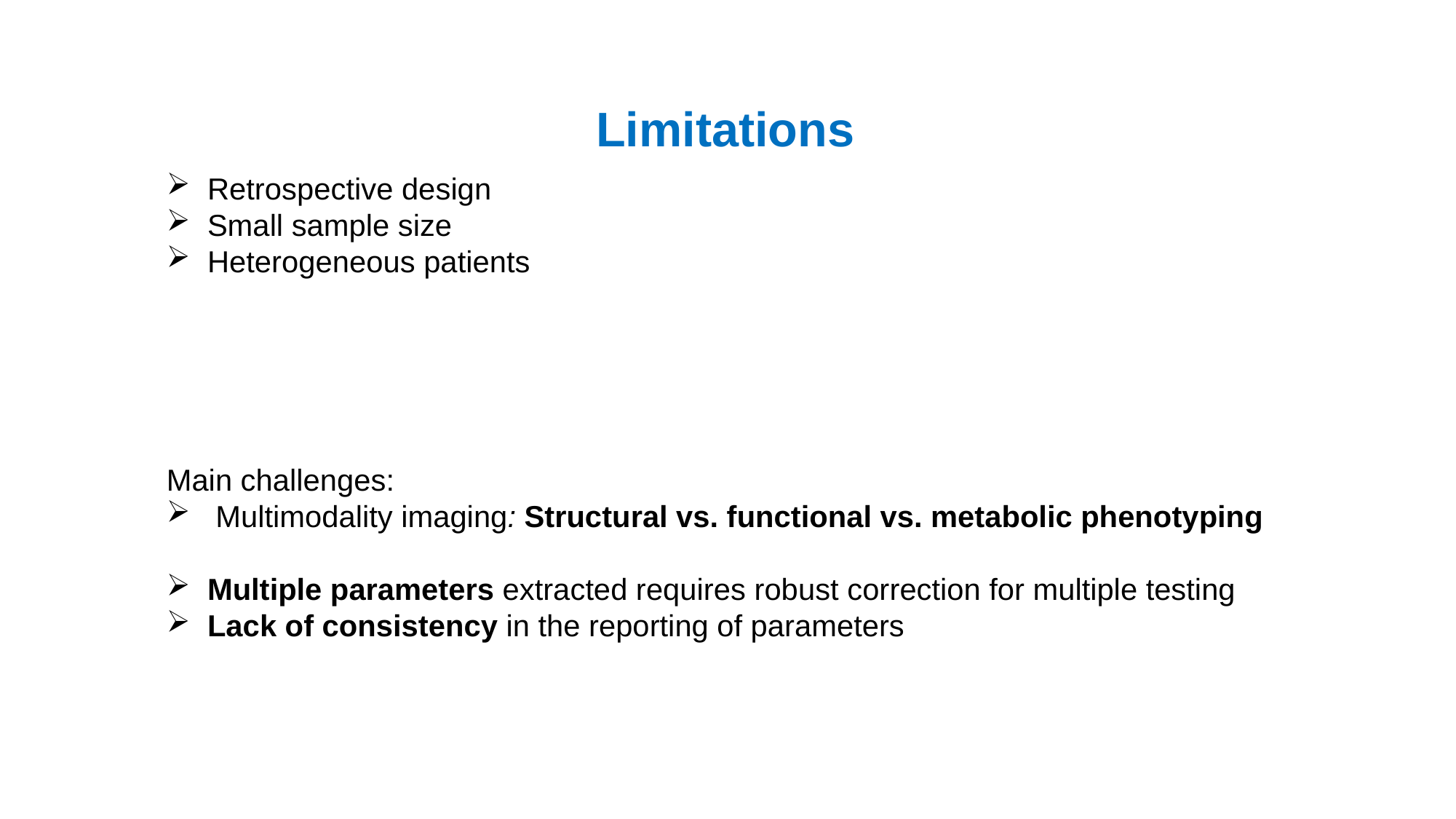

# Limitations
Retrospective design
Small sample size
Heterogeneous patients
Main challenges:
 Multimodality imaging: Structural vs. functional vs. metabolic phenotyping
Multiple parameters extracted requires robust correction for multiple testing
Lack of consistency in the reporting of parameters

## Slide 20
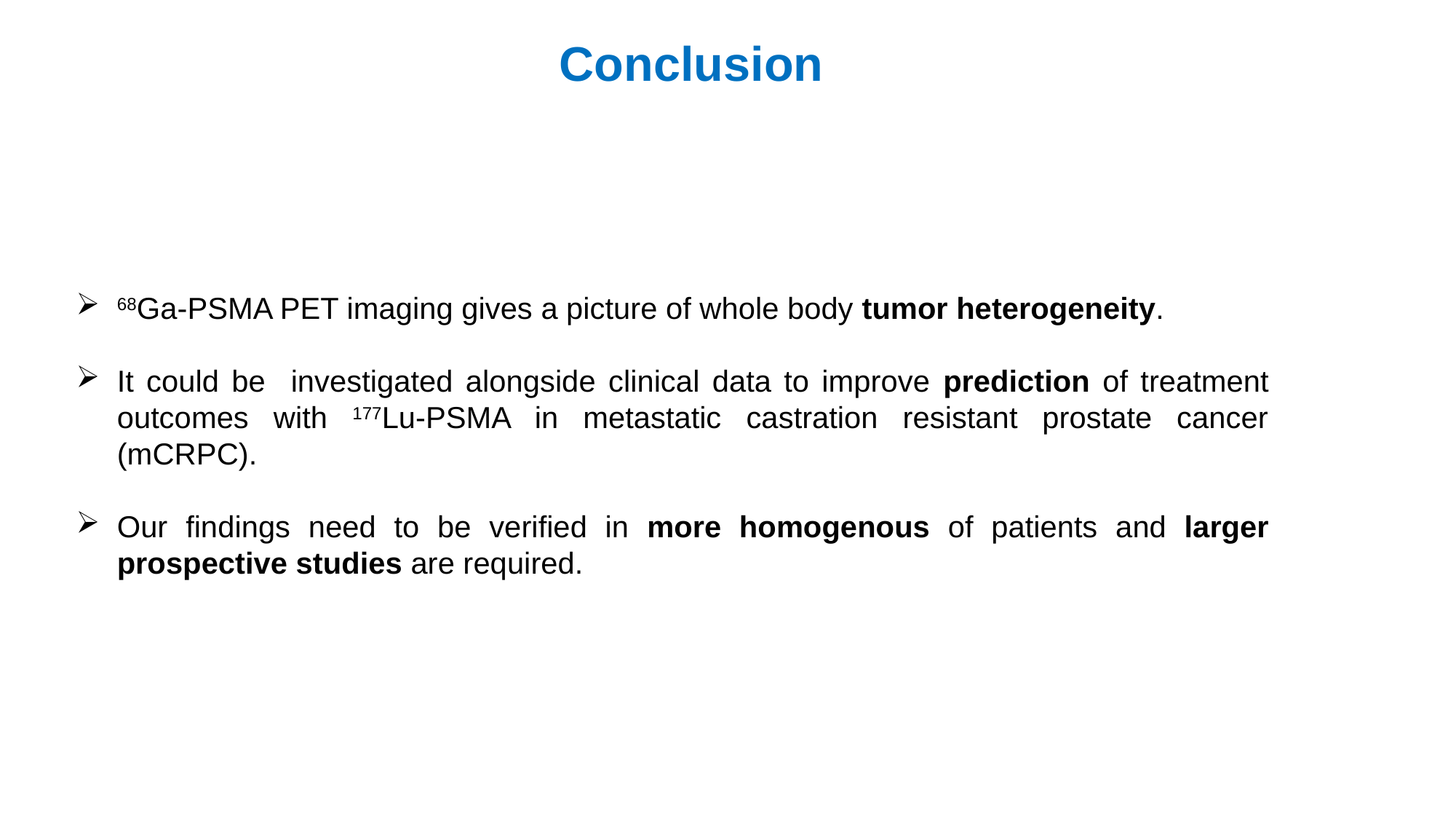

Conclusion
68Ga-PSMA PET imaging gives a picture of whole body tumor heterogeneity.
It could be investigated alongside clinical data to improve prediction of treatment outcomes with 177Lu-PSMA in metastatic castration resistant prostate cancer (mCRPC).
Our findings need to be verified in more homogenous of patients and larger prospective studies are required.

## Slide 21
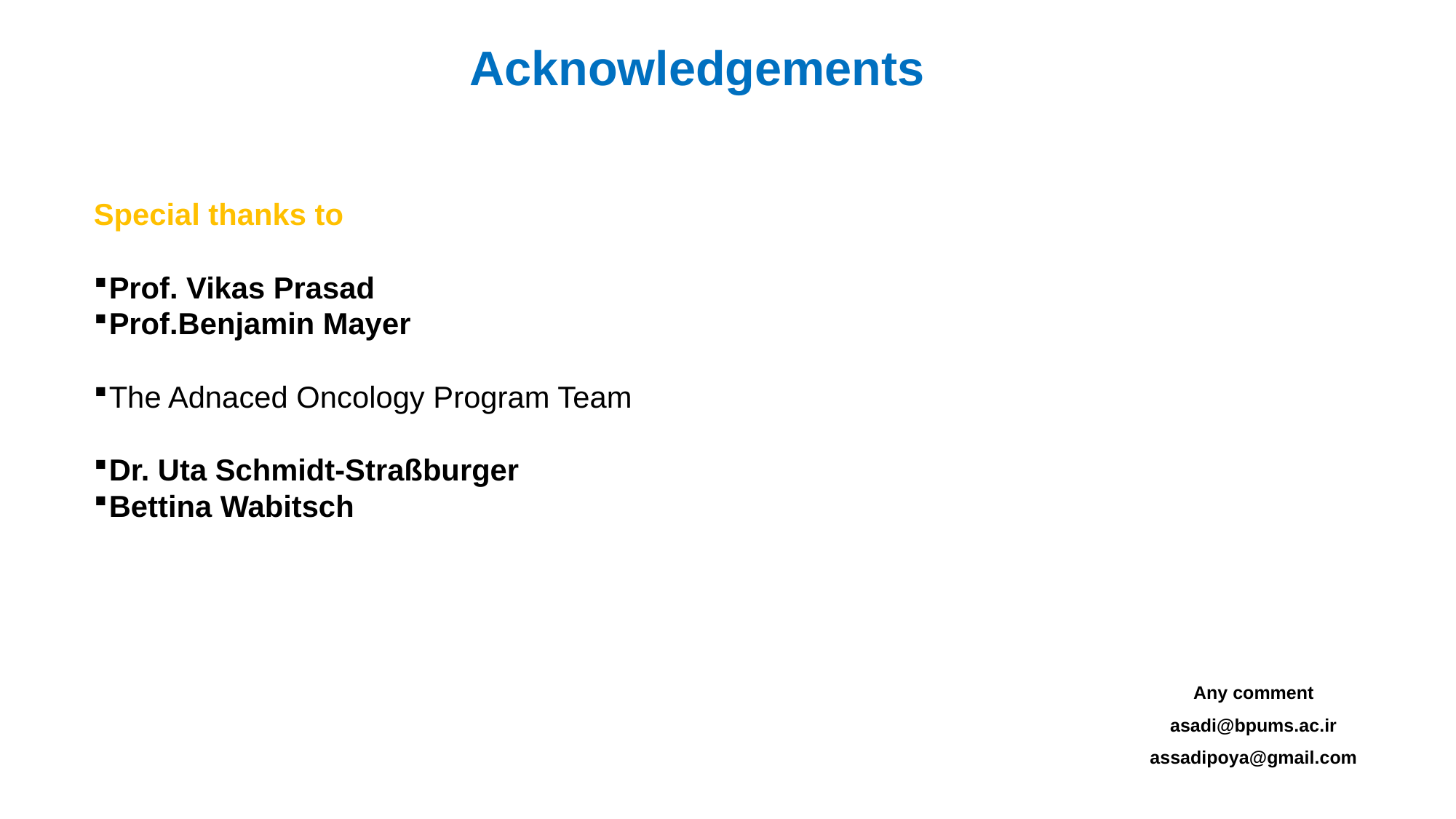

# Acknowledgements
Special thanks to
Prof. Vikas Prasad
Prof.Benjamin Mayer
The Adnaced Oncology Program Team
Dr. Uta Schmidt-Straßburger
Bettina Wabitsch
This
Any comment
asadi@bpums.ac.ir
assadipoya@gmail.com
